# Rapidly Screening the Correlation between the Rotational Mobility and the Hydrogen Bonding Strength of Confined Water

**DOI:** 10.1021/acs.jpcb.4c05397

**Published:** 2024-10-23

**Authors:** Alec A. Beaton, Alexandria Guinness, John M. Franck

**Affiliations:** Department of Chemistry, Syracuse University, Syracuse, New York 13210, United States

## Abstract

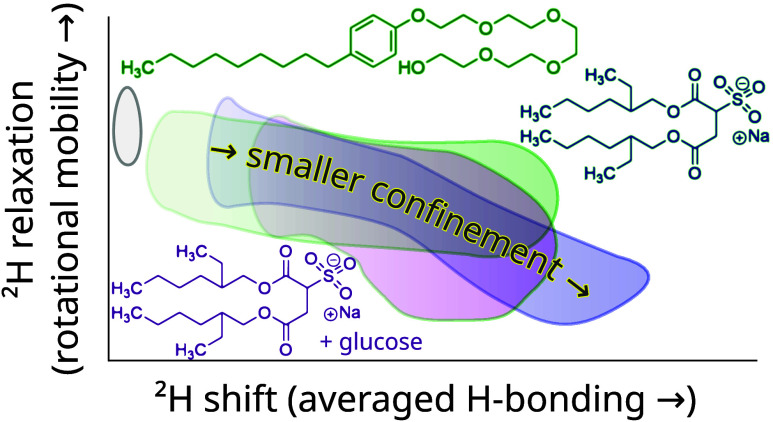

Automated Deuterium Relaxation-Ordered SpectroscopY in solution (ADROSYS), an automated two-dimensional deuterium NMR methodology, discriminates between D_2_O populations (as well as deuterium-labeled alcohol groups) whose properties differ as a result of being confined inside nanoscale volumes. In this contribution, a proof-of-principle demonstration on reverse micelles (RMs) yields the insight that as the length scale of the confinement decreases from several nanometers down to less than a nanometer, the position of the signal peak migrates through the two-dimensional (2D) spectrum, tracing out a distinctive path in the 2D space (of relaxation time vs chemical shift). The signals typically follow a relatively gentle linear path for water confined on the scale of several nanometers, before curving once the surfactants confine the water molecules to length scales smaller than 1–2 nm. The qualitative shape of this path, especially in the regime of strong confinement, can change with different choices of surfactants, i.e., a different choice of chemistry at the edges of the confining environment. An important facet of this research was to demonstrate the relatively wide applicability of these techniques by showing that both: (1) Standard modern NMR instrumentation is capable of deploying an automated measurement, even though the choice of a deuterium nucleus is nonstandard and frequently requires companion proton spectra in order to reference the chemical shifts; and (2) well-established (though underutilized) modern techniques can process the resulting signal even though it involves the somewhat unusual combination of chemical shifts along one dimension and a distribution of relaxation times along another dimension. In addition to demonstrating that this technique can be deployed across many samples of interest, detailed facts pertaining to the broadening or shifting of resulting signals upon inclusion of various guest molecules are also discussed.

## I Introduction

Water confined to nanometer-scale (yoctoliter and zeptoliter) volumes or pockets moves quite differently from bulk water. These changes in motion often correlate with changes in the freezing behavior of water (or lack thereof),^1−5^ changes in excess entropy,^6−9^ and changes in the types of chemical reactions that occur.^10,11^ Therefore, routine methods that can observe these alterations in water dynamics will soon provide insights into broad, general questions about the thermodynamics and phase behavior of the most important types of water molecules: those at interfaces and inside pockets. This is especially true if new, automated methods can offer insight into the properties and behavior of water from the perspective of the water molecules. Such methods offer the opportunity to understand how different solutes interact with a hydration layer [as modeled by a larger-diameter reverse micelle (RM)] or a macromolecular pocket (as modeled by a small-diameter RM) at a very powerful general level.

While fine distinctions exist among different modes of water diffusion,^12^ we can broadly classify them into two categories: First, several extant and emerging methodologies investigate the translational diffusion of water, i.e., thermal motions that displace water molecules by about a nanometer over tens of picoseconds to a nanosecond, necessarily involving the cooperation of many water molecules.^12−15^ Second, more mature methodologies examine the rotational diffusion of individual water molecules and have relied on various physical techniques to determine the time scale of such motions.^16−21^ As the capabilities of porous and interfacial macrochemistry progress, methods for interrogating rotational diffusion will prove both crucially important and complementary to methods for interrogating translational diffusion. Specifically, rotational motion, the focus of this publication, provides an excellent indicator of the presence of extremely slow-moving waters that could significantly affect the free energy if released into the bulk. Conversely, measurements of translational motion allow for systematic mapping of relative differences between patches of water located at different sites on a macromolecule.^13^

Deuterium nuclear magnetic resonance (NMR) relaxation sensitively reads out rotational motion, as the quadrupolar interaction of the deuterium nucleus tends to provide the dominant relaxation mechanism.^22−24^ For decades, researchers have exploited this capability to study samples using solid-state NMR techniques, notably in lipid systems,^25−27^ as well as to study variable properties of water molecules in other biologically relevant systems, such as DNA,^28^ glucose solutions,^29^ and elastin.^23,30^ In particular, the predominance of the quadrupolar relaxation mechanism in deuterium studies enables a more accurate measurement of intramolecular motion compared with proton NMR studies. This accuracy arises because proton NMR relaxation reflects not only rotational motion but also strong intermolecular dipolar interactions^21,31^ as well as slow-time scale processes such as chemical exchange.^28^

Here, the choice of RM samples enables a proof-of-principle measurement for a general methodology applicable to other systems; while RMs are relatively easy to prepare, the technique should apply equally well to other systems with trapped water, including solid mesoporous systems, porous and permeable polymer systems, and even biological systems with trapped or phase separated aqueous solutions. Much attention has been given to RMs from a variety of different communities. For instance, in materials science, different additives, such as polymers^32^ and cosurfactants,^33^ have been studied as a means to mediate certain properties of the system, such as droplet exchange and microemulsion stability.^34,35^ In biophysical sciences, work toward encapsulating proteins inside these systems initially found relevance in protein purification and extraction^36,37^ but is now used as a unique method for studying the proteins themselves,^38−40^ presenting myriad ways to prepare these systems to suit the needs of the research at hand. RMs can further serve as controlled environments for carrying out certain reactions,^41,42^ delivering biologically active molecules,^43^ or studying confinement effects on encapsulated molecules.^44^

A complication toward studying water in RMs often arises from the fact that water is typically only a small component of the overall mixture; in most cases, an inescapable amount of background signal arises from the dispersant and the surfactant and may complicate observation of the water signal. Therefore, the methodology proposed here focuses on deuterium NMR relaxometry, where D_2_O is used in place of H_2_O in sample preparation. Substituting H_2_O with D_2_O has been shown to minimally affect the properties of the Aerosol-OT (AOT) RMs despite differences, such as density and heat capacity, between H_2_O and D_2_O.^45^

This contribution focuses, in particular, on the fact that despite its promise, deuterium relaxometry has lagged in terms of taking advantage of modern data-processing methodologies and automation techniques. In particular, a few decades ago, Halle and others^4^ provided proof-of-principle demonstrations and several key applications,^28^ and these rigorous techniques continue to offer insight into the behavior of water.^5,23,24,46−49^ While such studies do seek significant rigor in the understanding of rotational dynamics, they tend to do so by relying on specialized instrumentation rather than capitalizing on the wide availability and automatability of modern commercial magnetic resonance instrumentation. The work of the Boutis lab^30,50−53^ demonstrates the utility of two-dimensional (2D) deuterium relaxometry on elastomeric peptides, and thus offers an important clue as to how this discipline might be advanced. Nonetheless, we still perceived a need for a simple, automated, yet powerful, measurement. Furthermore, we were not aware of methodologies that explicitly correlate the mobility measurements attained from quadrupolar relaxation with structural clues about the hydrogen bonding matrix that can be gleaned from changes in diamagnetic shielding.^54−57^ Therefore, in this article, we demonstrate a variant of 2D ROSY (relaxation-ordered) spectroscopy (analogous to diffusion-ordered DOSY, but replacing diffusive decay with relaxation decay). To achieve this, we deploy a standard “1.5 D Inverse Laplace Transform (ILT)” processing^58^ to display the correlation between the ^2^H NMR relaxation rate and the diamagnetic shielding (i.e., chemical shift). We crucially combine this methodology with customized Bruker automation software to open the possibility of rapidly screening tens or even hundreds of samples and refer to the result as Automated Deuterium Relaxation-Ordered SpectroscopY in Solution (ADROSYS).

## II Methods

### II.I Sample Preparation

**Figure 1:**
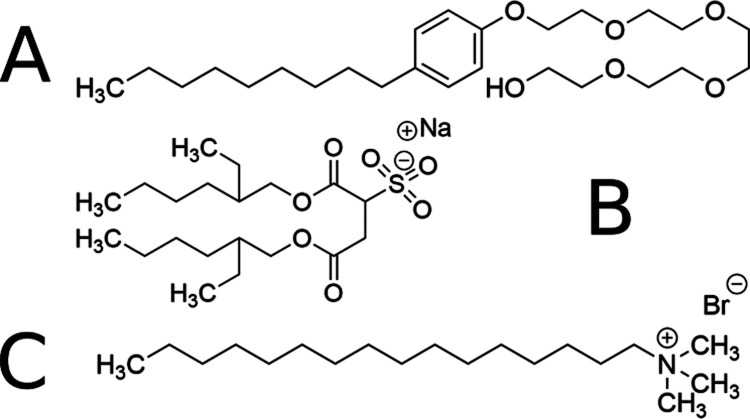
Three surfactants used in this work: (a) Igepal CO-520, (b) AOT, and (c) cetrimonium bromide (CTAB).

For the preparation of reverse micelles utilizing AOT (Figure 1) as the surfactant, equal volume samples of the lowest-*w*_0_ sample and highest-*w*_0_ samples (*w*_0_ = 1 and 20, respectively) were prepared from 2.82 g of AOT (6.3 mmol) and 141 mg of AOT (0.3 mmol), respectively, each in 6765 μL of dispersant, which was varied between isooctane (Alfa-Aesar), hexane (Fisher Scientific), and carbon tetrachloride (CCl_4_) (Sigma-Aldrich). Those that used Igepal (octylphenoxypolyethoxyethanol, Sigma-Aldrich) as a surfactant were prepared in a similar manner utilizing 2.86 g of Igepal (6.5 mmol) and 192 mg of Igepal (0.5 mmol), respectively, in place of the AOT, each in 6765 μL cyclohexane (Sigma-Aldrich). Reverse micelles containing CTAB (Sigma-Aldrich) followed a similar procedure using hexane as a dispersant but also included the alcohol cosurfactant 1-hexanol (Sigma-Aldrich) to stabilize the RM phase.^33,40,59−62^ Here, the highest *w*_0_ employed 0.360 mL (0.293 g, 2.87 mmol) of 1-hexanol per 0.116 g (0.318 mmol) of CTAB. The lowest *w*_0_ employed 0.460 mL (0.374 g, 3.67 mmol) of 1-hexanol per 0.116 g (0.318 mmol) of CTAB. Vortexing the desired surfactant and dispersant yielded a completely transparent solution. After subsequent addition of 115 μL (6.3 mmol) D_2_O (Cambridge Isotope Laboratories) to each solution, and an additional 3× vortexing for 30 s, samples sat at room temperature until completely clear. Perdeuterated hexane (38 μL, 0.29 mmol, Acros Organics) was added to each solution as a concentration and chemical shift reference.

Equal volumes of high- and low-*w*_0_ samples were mixed to generate a third sample. Subsequent samples were prepared by mixing two previously prepared sample mixtures, always using equal volumes. Thus, while mixing adjusts (*w*_0_ = *w*_0,1_^–1^ + *w*_0,2_^–1^)^−1^, the concentration of water per total solution volume remains fixed. After the addition of TMS (trimethylsilane, ∼1 μL, Thermo Fisher Scientific), each sample was vortexed for 3 × 30 s. In general, the thermodynamic stability of nanoemulsions can be identified by the turbidity of the solution^63^ and only clear and transparent solutions were analyzed here. NMR samples were then prepared by adding 600 μL of each mixture to separate 5 mm tubes and flame sealing.

For guest molecule studies, the same procedure was applied, except that first, a guest molecule stock solution of the desired concentration was made and 115 μL was used in place of the neat D_2_O. For the 49 w/w % poly(ethylene glycol) (PEG)-200 samples, the stock solution contained 1.049 g (16.9 mmol, Alfa-Aesar) of PEG-200 in 0.972 mL (1.07 g) of D_2_O. The 24 w/w % glucose stock solution contained 0.341 g of glucose (1.9 mmol, Fisher Scientific) in 1.00 mL (1.11 g) of D_2_O.

### II.II NMR Experiments

Data was collected using a Bruker Avance III HD 400 MHz spectrometer with a room-temperature smart probe. Deuterium experiments were run on the lock channel at 300 K. Preceding acquisition with autoshimming using a CDCl_3_ standard gave reasonable homogeneity, but automated gradient shimming (topshim) on the integrated proton free induction decay (FID) guaranteed routinely better homogeneity.

**Figure 2:**
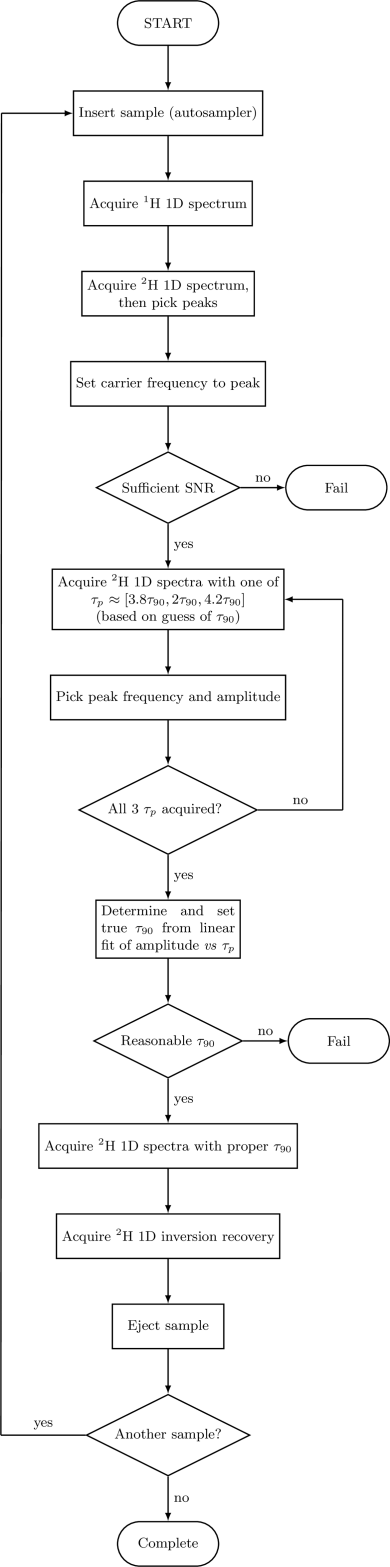
Flowchart illustrating the ADROSYS automation scheme. In particular, this automation scheme quickly but robustly optimizes the ^2^H pulse lengths for reliable *T*_1_ measurements and provides companion ^1^H spectra for accurate chemical shift referencing, despite variable solution susceptibilities.

When acquiring inversion recovery experiments, it is important that the pulse tip angles and exact resonance frequency are properly calibrated; here, reference one-dimensional (1D) spectra, both ^2^H and ^1^H (from TMS, surfactant, solvent protons, and residual HOD protons), were also desired. To automate data acquisition, an AU program was developed to acquire a single ^1^H scan, followed by a series of ^2^H scans to determine the optimal transmitter frequency and pulse width, in order to run an inversion recovery experiment with optimized parameters ( Figure 2). The automation program thus acquires all of the raw data needed for an ADROSYS spectrum, without any detailed input required from the user. It was also integrated into the IconNMR GUI, enabling multiple samples to be queued automatically.

Given the short (450 ms) *T*_1_ of ^2^H for D_2_O at room temperature (compared to 3.6 s for the ^1^H of H_2_O)^22^ compounded with the shortened relaxation times for water inside an RM, a maximum repetition delay of 1 s satisfies the requirement of 3–5× *T*_1_ for these RM samples. Typical inversion recovery experiments (customized pulse sequence^64^Section S10) involved 16 indirect steps with an 8-step phase cycle, acquiring 4096 complex points at 614 Hz spectral width along the direct dimension with only one scan per phase cycle step.

TMS provides a measure of the field strength (*B*_0_) in the ^1^H spectra taken immediately before the inversion recovery experiments. By calibrating the TMS peak in the ^1^H spectrum to 0 ppm, and using the relative magnitude of the gyromagnetic ratios for the ^1^H and ^2^H experiments, one is able to effectively reference the position of the ^2^H peaks relative to the field readout for the TMS peak (see Section S4 for more details). The resulting 2D NMR data were processed using code developed in house,^64,65^ with the ILT performed via adaptation of the Butler Reeds Dawson (BRD) algorithm.^58,66^ Whether the data is 1.5D or 2D, we utilize the basis set compression of ref (58) for greater efficiency.

**Figure 3:**
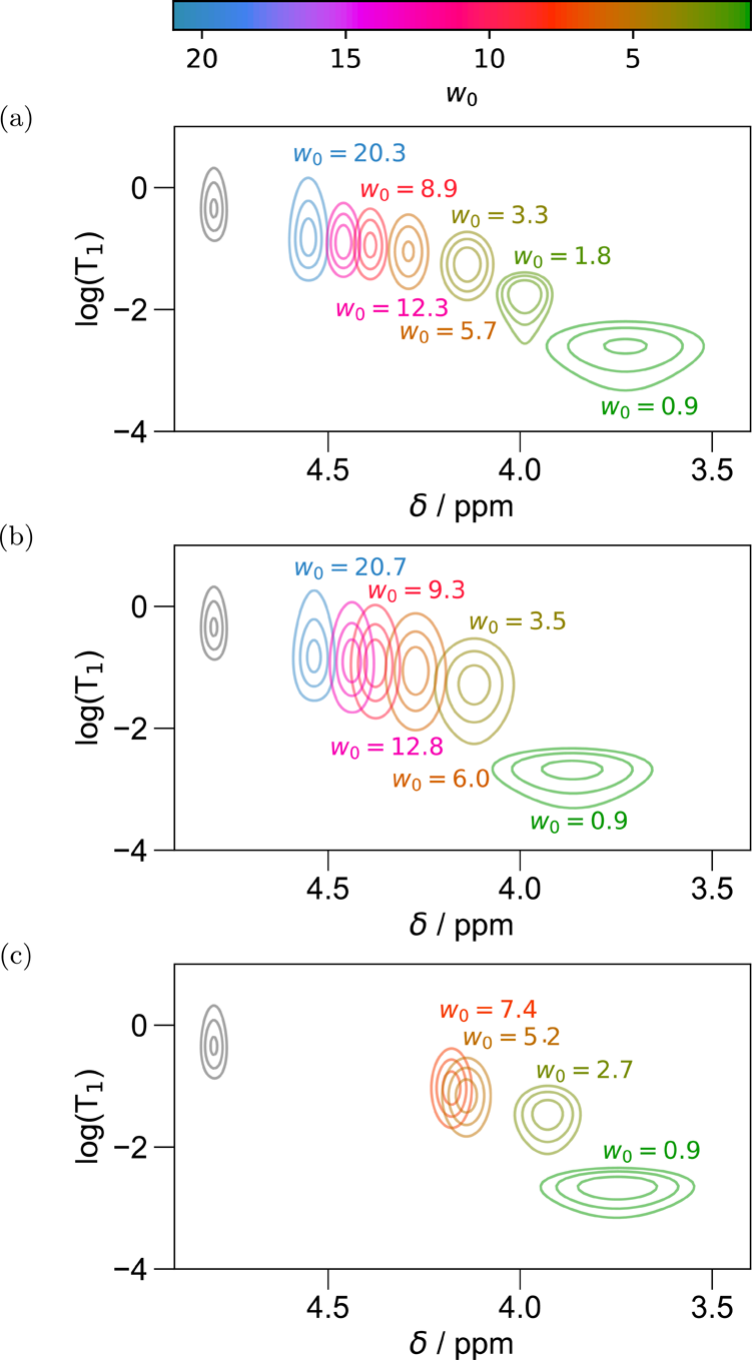
ADROSYS data illustrating the *T*_1_ vs chemical shift distribution for different AOT RMs prepared in hexane (a) and isooctane for water loadings ranging between 0.9 and 20.3 (b) and between 0.9 and 7.4 for CCl_4_ (c).

In order to present the contours for different water loadings (*w*_0_), the perceptually uniform “Lab” color space^67^ assisted in designing a custom colormap (Figure 3a, top). The vector distance of *L*, *a*, and *b* components in the Lab space corresponds to the accepted perceptual difference in the corresponding colors. This allows a choice of colormap where the variation in water loading values corresponds to the perceptual variation in the colors. Specifically, we choose the lightness (*L*) component to be the same for all water loading values, while the magnitude of gradient  in the color components *a* and *b* was set equal across the chosen colormap. Thus, to match the modernization of the data acquisition and processing, the details of the data presentation also capitalize on the widely available color-theory tools.

## III Theory

### III.I Reverse Micelles

While they serve as only approximate measures, two equations from the literature help to conceptualize the size of the RMs. The radius of the water pool (*r*, in nanometers) can be approximated by^56,68^
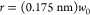
1where *w*_0_ gives the “water loading”—the molar ratio of water to surfactant. Considering an RM without guest molecules, the linear dependence of *r* on *w*_0_ is consistent with the basic consideration that, on the one hand, the volume (*V*) of the RM interior is proportional to *r*^3^, which is proportional to the number of water molecules per unit volume of solution, i.e., *V* ∝ *r*^3^ ∝ [H_2_O]; in contrast, surface area of the RM water pool is proportional to the number of surfactant molecules per unit volume of solution, i.e., *A* ∝ *r*^2^ ∝ [surfactant]. Various previous studies^69−71^ document the number of AOT (Figure 1b) molecules (aggregation number *n̅*) per RM, and therefore/alternately, the number of water molecules *n̅w*_0_. These studies demonstrate varying levels of agreement and therefore justify some amount of meta-analysis. For low-*w*_0_ samples, measurements of the osmolality of the RM aggregates from controlled-partial-pressure vapor-pressure osmometry (CPP-VPO) most accurately count the number of water molecules vs methods that rely in whole or part on the mass and/or density to determine the number of molecules per RM (especially, since the AOT molecule is almost 25× the mass of the water molecules). The CPP-VPO literature^71^ demonstrates a region with relatively constant *n̅*, followed by a *n̅* that grows linearly with *w*_0_. For large spherical RMs, one expects *n̅* ∝ *w*_0_^2^ (and number of water molecules *n̅w*_0_ ∝ *w*_0_^3^); as previously noted,^72^ two references^70,73^ employing velocimetry vs ultracentrifugation roughly agree on values of *n̅* for 15 < *w*_0_ < 30. Despite the significance of the work by Piletic et al.,^69^ we ignore the numbers they present for *n̅w*_0_, because they are implied to be rough estimates and the discrepancy with ref (70) is not explained. Digitization and nonlinear fitting of these data sets^70,71,73^ (Figure S1) yields an equation of the form
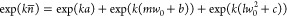
2with *a* = 15.1 controlling number of AOT molecules in the constant-*n̅* regime, *m* = 7.15 controlling the slope of the linear region (number of AOT molecules added per change in *w*_0_), *b* = 0.259 the intercept of the linear regime, *l* = 0.673 giving the curvature of the quadratic regime, *c* = −37.1 the intercept of the quadratic equation, and *k* = 0.174 controlling the curvature between the constant, linear, and quadratic regimes. Note that because the numbers for this fit come from digitized figures, the significant figures are not intended as a claim of accuracy but merely offer a means to reproduce the curve to estimate *n̅*. Furthermore, while the form of the equation roughly resembles an addition of concentration or equilibrium terms with free energies scaling as polynomials of *w*_0_, the rationale for the choice of functional form arose simply in order to generate a constant vs linear vs quadratic regime, in order to compile the *w*_0_ scaling noted in ref (71) vs ref (70).

Next, when guest molecules are incorporated, the definition of water loading (*w*_0_) can become ambiguous. Here, *w*_0_ is defined generally as
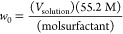
3where *V*_solution_ includes any guest molecules, and 55.2 M is chosen for the molarity of pure D_2_O (vs H_2_O which is 55.4 M). This was referred to as the “*w*_0_-equivalent” (as opposed to water:surfactant molar ratio) preparation in the work from Wiebenga-Sanford et al.^10^ To the extent that addition of aqueous solutes inside the RM surfactant shell does not perturb the density of aqueous solution, *w*_0_-equivalent RMs should adopt a similar size based on the *w*_0_ value, regardless of the identity (or absence) of guest molecules. However, Wiebenga-Sanford et al. found that RMs with guest molecules exhibit a slight size reduction relative to water-only RM preparations of the same *w*_0_, owing to a proposed greater spherical nature resulting from the osmolyte inclusion.

Finally, we note (from simple geometric considerations) that if a core–shell model can be assumed, then any property *x*_avg_ (whether chemical shift, relaxation rate, etc.) that presents as a weighted average of the core water and shell water will average as
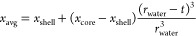
4where *t* is the thickness of the shell, *x*_core_ is the value of the property for water molecules in the core region, and *x*_shell_ is the value of the property for water molecules in the shell region.

### III.II ^2^H Relaxation

The rate of longitudinal relaxation, *R*_1_, of deuterium (total nuclear spin quantum number *I* = 1) in solution is dominated by the quadrupolar relaxation mechanism (Section S2), in contrast to relaxation of ^1^H nuclei , which is typically dominated by dipolar relaxation in solution.^31^ In the extreme narrowing limit (see Figure S2), where the correlation time τ_c_ is very fast relative to the resonance frequency ν_D_ (i.e., 2π*ν*_D_τ_c_ ≪ 1), *J*(ν) evaluated at frequencies of ν_D_ and 2ν_D_ will equal 2τ_c_. This condition is applicable to the overwhelming majority of samples in this work, and simplifies eq S1 to
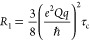
5through which we can relate the rotational correlation time of the deuterium nuclei, τ_c_, to the corresponding *R*_1_. Notably, under the extreme narrowing limit, *J*(0), *J*(ν), and *J*(2ν) all evaluate to 2τ_c_ rendering the value of eq S3 equal to that of eq 5. In other words, in the motional narrowing limit (and only in the motional narrowing limit), *R*_1_ = *R*_2_, where *R*_2_ is the transverse rate of relaxation.

When considering relations to other techniques, it is important to note that the τ_c_ value above can represent the net effect of several individual processes or environments. When multiple processes contribute to the rotational diffusion, the rate of decay of the quadrupolar correlation function is additive, i.e., the total correlation time takes the form
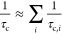
6where the individual τ_c,*i*_ give the rotational correlation times of the individual processes. Note that the simultaneous presence of shorter time scale motions suppresses the effect of longer time scale motions. For example, if the correlation function, which physically represents the persistence of the water molecule orientation here, decays due to both the tumbling of the water molecule relative to the entire RM, as well as due to the tumbling of the entire RM, then the shorter of the two correlation times will dominate, as indicated by eq 6. Similarly, in the unlikely event that the molecules exchange, on a time scale faster than the correlation time, between environments where they have different mobilities, then the decay of the correlation function will average between the two environments, as indicated in eq 6. This corresponds to the averaging of the inverse of the relaxation rates or to an averaging of the relaxation times (*R*_1_^–1^ = *T*_1_).

On the other hand, in the more likely event that molecules exchange between environments on a time scale longer than τ_c_, but still shorter than the time scale of the ^2^H relaxation (*T*_1_ = *R*_1_^–1^, typically order of tens of ms), their relaxation rate would average. Following from eq 5, this means an observation of τ_c_ via the relaxation time would report an average:
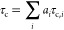
7where the individual τ_c,*i*_ give the different rotational correlation times that the water molecule adapts in each particular environment, and the *a*_*i*_ give the fractional amounts of time for which the molecule resides in each environment. Notably, because eq 7 relies on eq 5, it is valid only in the limit that all τ_c_ fall in the motional narrowing regime.

Importantly, ^2^H measurements that observe a single relaxation time do not rule out the possibility of fast exchange between environments with different correlation times. Conversely, if a distribution is observed with more than one relaxation time, such an observation indicates the existence of two populations of water that remain separate on a time scale similar to or greater than the relaxation time.

## IV Results and Discussion

The new automated technique presented here enables a systematic study of the water pools in RMs as a function of *w*_0_, for RMs prepared with different surfactants, different dispersants, and different additives, monitoring each system for evidence of correlated changes in the rotational mobility and average hydrogen bonding strength. In particular, it explores a series of different RM samples in order to test the hypothesis that distinct and noticeable changes in the pattern of correlated rotational mobility and hydrogen bonding will occur as the size of macromolecular structures (here the size of the RM water pool) crosses length scales comparable to the correlation length of water.^3,74,75^

RMs are thermodynamically stable mixtures comprising droplets of water encapsulated by surfactant and dispersed in an apolar medium. As a demonstration system for this technique, RMs offer a unique opportunity to construct water pools with a continuous range of different sizes while keeping the chemistry at the outer edge of the pool the same for all sizes. One can rapidly generate a wide range of RMs without advanced synthetic capabilities, and they serve as useful nanocontainers for chemical reactions as well as adjustable and controlled model systems for the study of confinement. The water loading also correlates to the size of the water pool, with sizes ranging from less than 1 nm to slightly more than 10 nm at low (*w*_0_ = 1) and high (*w*_0_ = 60) water loadings, respectively.^56,68^ The water pools inside these RMs have been studied for several decades by a range of techniques, including NMR,^21,54,76^ neutron scattering,^77,78^ light scattering,^79^ dielectric relaxation,^80,81^ infrared spectroscopy,^69,82−84^ and ESR.^85−88^ These various methods all offer support for a general increase in the bulk-like nature of the water within this water pool as the water loading (i.e., *w*_0_, water to surfactant molar ratio) increases. However, frequently, studies may be confined to one type of RM system at a time or else focus on RMs prepared only with a subset of possible *w*_0_.

### IV.I Trend of Correlated Measurement vs *w*_0_

An increase in *w*_0_ induces a correlated increase in chemical shift and *T*_1_ in all cases (hexane, isooctane, and CCl_4_), as shown in Figure 3a–c. The order of magnitude of *T*_1_ increases rather sharply from the lowest water loadings up to *w*_0_ ∼ 3–5. At *w*_0_ ∼ 3–5, the signals transition into a regime of gentle increase of *T*_1_ that extends up to the highest water loadings. Both the diamagnetic shielding and the *T*_1_ approach, but never quite reach, the bulk values. (AOT preparations were also stable to higher *w*_0_ than shown here, and acquired, but these merely extend the trend seen here, and cannot be compared to other surfactants where such high *w*_0_ cannot be achieved.) Furthermore, a qualitative extension of the observed trend appears to closely, but never quite exactly, converge on bulk water. This serves as a reminder that even the largest RM water pools are small on a bulk scale and may be subject to effects, including but not limited to pH modulation by the surfactant headgroups, different rates of proton exchange, local variations in the magnetic susceptibility, or nonspherical shape distributions, all of which might contribute slight variations to the diamagnetic shielding or longitudinal relaxation.

An extremely popular and widely studied surfactant, AOT readily forms RMs in a three-component mixture of surfactant, dispersant, and water.^57,63^ In the literature, it has not been atypical to develop results and conclusions from data that focus entirely on RMs in the higher water loading regime that here corresponds to the regime of a gentle increase in *T*_1_. Previous results support the trend in D_2_O *T*_1_ measurements in this regime, both in AOT^21^ and in other surfactant systems.^89^ However, the data of Figure 3a–c highlight the value of expanding this viewpoint both to observe the correlation of chemical shift and *T*_1_, as well as to span the entire range of thermodynamically stable preparations of varying *w*_0_, including low *w*_0_ values.

We propose that these measurements lend credence to the idea that the chemical/physical environment inside the RMs truly undergoes a crossover between the two limiting cases. The overall shape formed by the collection of signals from samples with different *w*_0_ can vary significantly for different surfactant/dispersant combinations and therefore proves to be one of the most dramatic observables provided by ADROSYS. At the extreme of low *w*_0_, identified here by the regime where the log(T_1_) and chemical shift demonstrate a strong negative correlation, the shape traced out by the collection of signals from samples containing AOT with different *w*_0_ (any of Figure 3a–c) curves sharply downward. In this low-*w*_0_ regime, individual water molecules interact tightly with the surfactant via hydrogen bonding or charge-dipole interactions. The correlated change in chemical shift and *T*_1_ tells a consistent story indicating that confinement leads to a dramatically nonbulk-like environment where hydrogen bonds are possibly broken and where rotational dynamics are slowed. At the extreme of high *w*_0_, identified here as the regime of more gently increasing *T*_1_ and chemical shift, waters are expected to interact overwhelmingly with other water molecules via hydrogen bonding, leading to more “bulk-like” behavior. The interactions with other water molecules are expected to be cooperative in nature, while the interactions with the surfactant are potentially far more localized. It is worth noting that the crossover between these two regimes corresponds roughly to important RM size thresholds in other studies, such as water loadings where confined water does not freeze (*w*_0_ ≈ 6.5), and where other dramatic effects of confinement are evident.^2,90,91^ It is also worth noting how this points to the value of a strategy not of attempting extreme rigor in the interpretation of individual measurements but rather in employing modern technology to automate many measurements and to observe the correlated changes of more than one measurement.

### IV.II Effect of Different Dispersants

The identity of the dispersant is also an important factor in determining the stability of RM systems.^63^ Careful combination of dispersants provides a nuanced control of phase stability,^92^ emphasizing the complex relationship dependence of RM stability on dispersant. Dispersant choice also has been shown to impact the shape of RMs,^93^ and such shape fluctuations have been shown to complicate data interpretation and analysis.^94^

Despite the ability of isooctane to accommodate a larger *w*_0_ than hexane before turning into (thermodynamically unstable, i.e., non-RM) emulsions,^63,95^Figure 3b shows that up to a *w*_0_ of approximately 20, the correlated measurement reports no dramatic difference in the trend in mobility vs hydrogen bonding environment of the water pool when suspended in isooctane vs hexane. Thus, these data would support the result that free energy contributions from the water itself likely cannot rationalize the differing phase stability and that interactions between the solvent and surfactant likely drive the differences in stability of the two systems, supporting molecular dynamics (MD) results that water energies do not drive the formation or structure of the RMs.^72^ (Likely, differences arise from the ability of each to support different surfactant curvatures.)

This is not to say that there is no observable difference upon a change of dispersant: RMs prepared with equivalent *w*_0_ in the different dispersants do display some differences in the chemical shift positions. In particular, while maintaining a qualitatively similar 2D correlation pattern, the range of chemical shifts of D_2_O inside RMs dispersed in hexane expands relative to those from RMs dispersed in isooctane. The chemical shifts of *w*_0_ = 1 and *w*_0_ = 1.9 correspond to slightly greater magnetic shielding in hexane vs isooctane, which could suggest slightly weaker average hydrogen bonding, while at *w*_0_ ≈ 20, the opposite is true. Meanwhile, CCl_4_ shows peaks with more shielding (relative to both isooctane and hexane) for all *w*_0_, suggesting that the water pool has a more frustrated hydrogen-bonding matrix. While it is possible that the suggested changes in hydrogen-bonding could provide insight into the change in the stability of the RMs, it is also possible that they arise from differences in ellipticity of the different RM systems; from stronger electric fields at the CCl_4_ RM interface (due to interactions between the surfactant and CCl_4_); or from reduced intercalation of CCl_4_ into the surfactant layer that leads to less shape fluctuation and longer-lived water/AOT interactions that in turn reduce the ability of water to hydrogen bond with other water molecules. Finally, it is important to acknowledge that changes in average hydrogen bonding strength or duration might not be the only reasons for changes in chemical shift, especially in smaller RMs; differences in polarizability may arise from more detailed changes to the motion of electrons within the sulfate groups, etc. Therefore, we will interpret changes in chemical shift broadly as a “frustration” of the hydrogen-bonding matrix, simply reflecting that effects leading to changes in the magnetic shielding at the deuteron (or even the presence of ring currents, etc.) correspond to an alteration away from the transiently hydrogen-bonded structures typically formed by bulk-like water.

It is known that CCl_4_ cannot support *w*_0_ larger than approximately 10,^83^ owing to its high polarizability, leading to strong interactions between it and the surfactant headgroup dipole.^57^ In spite of this, CCl_4_ continues to be widely used for RM studies due to its spectroscopic silence in important measurements, such as IR spectroscopy,^84,96,97^ neutron scattering,^77,97^ and dielectric relaxation.^80,97^ The series of signals from samples with different *w*_0_ clearly trace out a distinctive shape in the 2D spectra of Figure 3a,b. Comparing that shape and its location to the signals in Figure 3c clearly indicates that all stable CCl_4_ RMs fall in the regime where the signals in the ADROSYS plot of Figure 3a,b trace out a shape that curves strongly downward. That is, even the largest CCl_4_ RMs that can be made have not yet entered the regime in which the rotational motion changes gently as a function of the hydrogen bonding strength. One notable interpretation of this result is that CCl_4_ RMs never enter the regime where they can be thought of, even approximately, as possessing a separate hydration layer “shell” and more freely moving water “core”; rather all values of *w*_0_ possible with CCl_4_ dispersant likely correspond to all water molecules experiencing some level of confinement. As various seminal infrared and dielectric studies^80,81^ utilized the CCl_4_ system as a model system, the interpretation of those studies would be likewise colored by the realization that all the water under study was subject to what appears here as identifiable confinement.

### IV.III Effect of Different Surfactants

The choice of surfactant has been shown to impact the behavior of the RM water pools, with differences noted in the behavior of water in AOT, an anionic surfactant, compared to Igepal, a neutral surfactant (Figure 1a).^98^ To further complicate comparative measurements, some surfactants such as CTAB (cationic, Figure 1c), or Triton X-100 (neutral) typically are used in the presence of cosurfactants,^60,99^ or particular dispersant preparations,^98,100^ where these choices in sample preparation usually center around the desire to achieve higher water loadings while still maintaining thermodynamically stable RM microemulsions. Small differences in the surfactant structure, such as the number of ethylene oxide units in Triton X-100, can additionally alter the stability of RM preparations.^101^

ADROSYS can also interrogate how the water matrix responds to substitution of various surfactants: anionic AOT surfactant vs neutral Igepal CO-520 vs cationic CTAB surfactant. The literature guides the experiments in the choice of a typical dispersant for each surfactant: isooctane for AOT,^76,102,103^ cyclohexane for Igepal,^104,105^ and hexane for CTAB.^62,106,107^ Additionally, for the CTAB surfactant, an added single-chain alcohol cosurfactant (here, hexanol, though other choices are available^33,40,59−62^), acts to stabilize the RM phase.

As compared to water in the AOT systems, the CTAB water exhibits similar chemical shifts and *T*_1_ correlations for the same *w*_0_ values (Figure 5), in spite of the opposite charge of the headgroup and the presence of the uncharged alcohol cosurfactant.

Notably, CTAB RMs yield two deuterium resonances for *w*_0_ above 12, which converge to a single resonance below *w*_0_ ≈ 12. The lower intensity, more shielded (lower ppm) resonance likely arises from a deuterated alcohol group generated by exchange between the D_2_O and the hexanol cosurfactant. Because the two peaks have ∼10 different intensities, Figure 5b shows this hexanol resonance (with the higher ppm range signal set to zero), whereas Figure 5a shows the D_2_O peak (with the lower ppm range signal set to zero).

The hexanol peak has a relaxation time shorter than that of D_2_O for the same *w*_0_, which is consistent with the expectation of very slow exchange and very slow rotational correlation times for the nuclei at this position. As *w*_0_ increases, *T*_1_ of the hexanol peak also increases, though only slightly. Given that the hexanol resides at the interface of the surfactant and RM, these observations suggest that there is more mobility at this interface for larger *w*_0_, which may be an important factor toward deciding the phase stability of the system. Interestingly, the hexanol resonance exhibits increased shielding (lower chemical shifts) as the *w*_0_ increases—anticorrelated with the effect observed for the D_2_O resonance. Thus, as the water pool grows in size, the increase in D_2_O chemical shift suggests a decrease in the frustration of the internal water pool’s hydrogen bonding network. At the same time, the decrease in the chemical shift of the hexanol resonance with an increase in *w*_0_ suggests a decrease in the hydrogen bonding strength of the hexanol group. Such an effect likely represents a “decoupling” of the hydrogen bonding matrix of the water from the alcohol groups at the surface. As the *w*_0_ increases, the opportunity for water molecules to hydrogen bond to other water molecules improves, while at low *w*_0_, the hexanol appears to be more strongly hydrogen bonding to the internal water pool. As the *w*_0_ increases and the water pool grows in size, the role of hexanol in hydrogen bonding to the water becomes less pronounced, providing an interesting insight into the role of this cosurfactant.

In general, these hexanol results also point to the capability of this technique to resolve multiple types of deuterons, when they are present in distinct populations that do not exchange on the time scale of *T*_1_. In that sense, these results also emphasize that because the deuterons in all of the other samples studied here yield a single peak in the ADROSYS spectrum, they must be present in water molecules that either share very similar properties or else exchange on a time scale faster than *T*_1_ (here, tens to hundreds of ms). In other words, the measurements of all other systems actively agree with the typical picture of nanoemulsions as thermodynamically stable systems with uniform populations of aggregates (i.e., RMs) that all present similar-sized pools of water with similar properties.

### IV.IV Choice of Surfactant Can Induce Qualitative Differences in Water Properties

**Figure 4:**
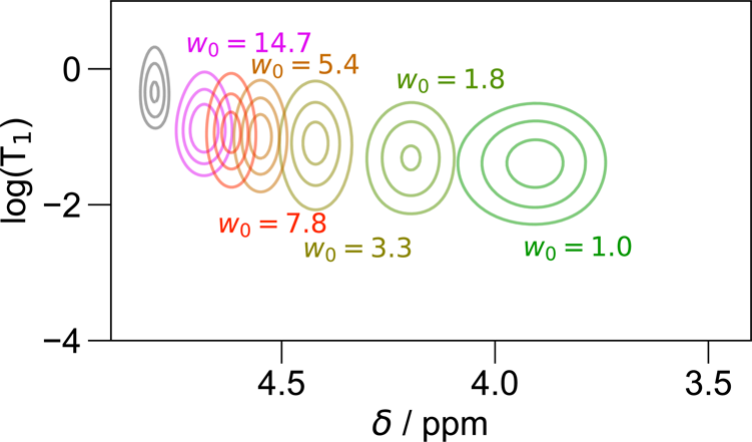
ADROSYS data illustrating *T*_1_ vs chemical shift distribution of D_2_O for different Igepal RMs prepared in cyclohexane for water loadings ranging from 0.9 to 20.3.

Overall, the surfactant appears to exhibit a greater effect on the water dynamics than did the dispersant. The importance of surfactant composition is underscored by the significantly different correlation between the chemical shift and *T*_1_ values observed for Igepal (Figure 4) vs the other surfactants (esp. Figure 3) studied here. As shown in Figure 4, the signals, overall, move up and to the left relative to Figure 3. Given these increases in both *T*_1_ and chemical shift relative to AOT in hexane or isooctane, the water in Igepal RMs appears to be engaged in more bulk-like hydrogen-bonding and rotational motion. The switch from sulfate to polyethoxylate headgroups stabilizes more “bulk-like” behavior of water at room temperature.

Perhaps most noticeably, the results show a qualitative difference in the shape of the trend traced by the signal as a function of *w*_0_: While (as previously discussed) the shape traced out by the collection of signals from water encapsulated inside AOT (Figure 3) curves downward at low *w*_0_, the trend in Igepal (Figure 4) shows that the correlation between the *T*_1_ and chemical shift rather flattens out, ultimately exhibiting changing chemical shift despite relatively constant *T*_1_. In the case of Igepal, the presence of the bulky polyethoxylate groups would be expected to alter the dynamics and transient structures of the water at the lowest water loadings, with the polyethoxylate side chains contributing additional hydrogen bond acceptor sites. Notably, there is an increase in the *T*_1_ by an approximate order of magnitude and the line width is correspondingly halved, from 0.9 ppm in AOT/isooctane to 0.4 ppm in Igepal. The increased mobility may arise from the ability to more quickly jump between the larger variety of H-bond acceptor sites available (as compared to the comparatively limited sites offered by AOT’s sulfate). Meanwhile, the chemical shift still indicates that, like for AOT, the hydrogen bonding matrix remains frustrated in the confined environment, likely since the ethoxylate groups do not also provide additional H-bond donor sites. The different curvature observed here, likely arising from the combination of the increased mobility and unresolved frustration, importantly emphasizes that the water pool inside very small Igepal RMs presents very different properties vs the pool inside small AOT RMs.

**Figure 5:**
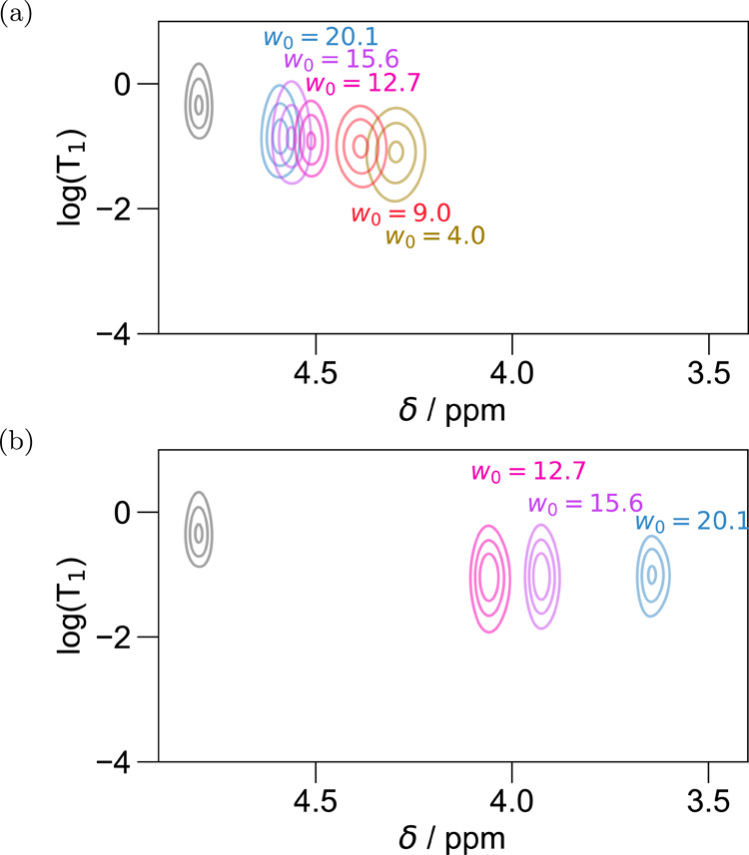
Results for CTAB with hexanol cosurfactant in hexane for the (a) D_2_O resonance and (b) hexanol resonance, which is observed in the samples above water loading 12. The lower plot is 10× less intense than the upper plot. Molar ratios of hexane to CTAB and hexanol to CTAB are 102 and 7.8, respectively, for the lowest *w*_0_ and 152 and 9.1, respectively, for the highest *w*_0_.

The straight aliphatic chain of CTAB does not support the high curvature of smaller reverse micelles and only forms a stable RM phase for *w*_0_ greater than about *w*_0_ ≥ 5. This obviously hampers the ability to compare CTAB to other surfactants across lower *w*_0_ values, where the obvious, qualitative differences are evident when comparing the signal from AOT vs Igepal RMs. Because of this, the data neither support nor invalidate the hypothesis that the unexpected mobility of the water in low-*w*_0_ Igepal RMs arises from the presence of the PEG-like groups, versus the hypothesis that it simply arises from the lack of charge of the headgroups. On the other hand, at higher *w*_0_, noting that the correspondence of data in Figure 5 with those in Figures 3b and 4 can be ascertained from the color scheme (which is identical for identical *w*_0_), comparisons can be made. While the results do indicate some differences in average hydrogen bonding strength, the mobility and overall position in the 2D correlation plot are similar for CTAB and other charged surfactant systems.

Overall, these results that compare the effects of different surfactants highlight the extremely close-range interactions between water and the RM headgroups and may arise from two independent causes. In the first case, they may indicate that interactions between water and charged AOT headgroups restrict the water motion and that relieving this interaction improves the mobility. AOT is a sulfonate with extremely low p*K*_a_. The sulfonate restricts the water,^108^ and is present in the RM core in very high local concentrations. In fact, for context, at even the highest *w*_0_ values employed here, a similar ratio of sodium sulfate to water would constitute a supersaturated solution. Conversely, it is possible that the ethoxylate units in Igepal contribute to an expansion of the water hydrogen bonding matrix, thereby effectively reducing the level of confinement. The polyethoxylate portion of Igepal resembles a PEG chain, which engages in particularly fruitful hydrogen bonding with the water matrix.^109^ The molecular weight of this chain is 237 Da. At lower water loadings, the mass of polyethoxylate inside the core of the RM likely exceeds that of the water. Even though PEG has been hypothesized to encourage an “iceberg-like” cage structure of water molecules,^110^ it could still be acting here to effectively expand the size of the water pool.

### IV.V Core–Shell vs Strong Confinement

The water inside RMs is known to exhibit different properties depending on the water pool size, ranging from very restricted motion at small sizes to a more typical, rapid, bulk-like motion at larger RM sizes.^111^ These water pools are accepted to follow a core/shell structure, with water in the core exhibiting more bulk-like properties and water in the shell exhibiting properties of interfacial water with restricted motion.^69,112^ Notably, however, the ability to quantitatively determine the properties of water based on a weighted sum of core and shell molecules is generally assumed to be valid only above a certain *w*_0_. Further complicating the picture, different techniques point to different RM sizes (values of *w*_0_) at which the bulk-like properties arise or become dominant. For example, dielectric relaxation dispersion studies have indicated this bulk-like crossover occurs above^80^*w*_0_ = 6, whereas ESR indicates bulk-like dominance above^86^*w*_0_ = 10, and established dominance above^88^ 20. IR data indicates bulk-like dominance above^113^*w*_0_ = 16.5, yet IR supplemented with MD points to^90^*w*_0_ = 7.5. Meanwhile, even differential scanning calorimetry (DSC) offers input here, demonstrating that the water inside RMs of *w*_0_ ≥ 7 demonstrates a freezing transition when cooled into what is now known as “no man’s land,” while water inside smaller RMs demonstrates no such transition.^2^

Overall, the correlated trends observed here highlight the truly unique properties of water inside RMs with very low (*w*_0_ < 6) water loadings. Water inside very small RMs is not simply interfacial water, but experiences additional confinement effects, here termed “strong confinement.” Notably, the strong confinement effects arise as the length scale of the water pool approaches the correlation length of water (∼1 nm). While higher water loadings demonstrate relaxation times and chemical shifts that converge close to the value of bulk water, as noted previously, one of the most distinctive features uncovered by the ADROSYS measurements here is the trend of the signals as a function of *w*_0_. In Figure 3, these display a dramatic change in slope near *w*_0_ ≈ 3–4. Recalling that the *y*-axis here represents roughly the logarithm of the rotational correlation time, this change in slope represents the point at which the water mobility effectively approaches zero.

Weighted sums of water populations in two separate environments, e.g., a core of “bulk-like” water and a shell of surfactant hydrating water, have been shown to be capable of explaining the properties of larger RMs. Attempting to fit such models to ADROSYS data that include very small *w*_0_ RMs proves to be an insightful exercise. In the first such model, water molecules exchanging between two environments on a time scale longer than the rotational correlation time, τ_c_, would display an averaged rate of relaxation; because the rate of relaxation is proportional to τ_c_ (eq 5), this corresponds to an averaged τ_c_. Attempts to fit the correlated chemical shift and relaxation rates to such a core–shell model failed to find a reasonable interpolation between different correlation times (and relaxation rates) via eqs 4 and 1—as the general form of the trend does not match the data, regardless of the choice of parameters. In fact, as shown in Figure S3, the curvature of trend traced by the correlated signals generated by such a model is opposite the curvature demonstrated in the correlated trend for all surfactants except Igepal.

A model where the *T*_1_ varies linearly with the fraction of core vs shell water molecules does generate a trend with the same curvature as seen in AOT samples (Figure S3, filled circles). The resulting fit cannot support a linear interpolation of both diamagnetic shielding and *T*_1_ based on an averaging of core and shell waters that can explain the behavior of very low water loading RMs; however, the general form of the resulting curve matches the data far better than a model that assumes the averaging of *R*_1_ (and τ_c_). Importantly, as noted previously, a core–shell argument for averaging of *T*_1_ ∝ 1/τ_c_ likely lacks physical motivation, requiring as it does exchange of water molecules between environments on a time scale faster than τ_c_. One could propose that the trend arises from changes to the collective network of water that roughly interpolate the rate of decay of the rotational correlation function (i.e., 1/τ_c_) in a fashion that scales with the RM size. This potentially includes an overall slowdown of all rotational motion of the water molecules and ultimately, at the lowest *w*_0_, leaves only a correlation function decay driven overwhelmingly by the rotation of the entire RM.

Overall, as previously noted, the shape traced out by the collection of signals arising from water confined inside AOT curves strongly downward at very low *w*_0_. One can propose that this feature signifies the transition between RMs that contain more clearly distinguished core (more bulk-like) vs shell (interfacial) water to RMs exhibiting strong confinement, where all of the water molecules undergo some level of confinement effect. While it has been widely shown that at larger length scales, a simple weighting between interfacial and bulk-like water can explain many measurement results, the strong confinement regime proves particularly interesting. The strong confinement can be explained in terms of reducing the water pool to a size smaller than the interfacial shell or simply in terms of reducing the total number of options (microstates) for different favorable hydrogen bonding configurations by decreasing the number of water molecules. In either case, the progressively more dramatic differences in the behavior of the water in the strongly confined regime emphasize the collective nature of water motions, which require hundreds (eq 2) of water molecules even to yield measurements that would fit nicely to a core–shell model. Furthermore, the ADROSYS measurements here also illustrate two significantly different signal patterns (comparing Figure 3b vs Figure 4) in the strongly confined regime associated with ionic vs nonionic surfactants.

### IV.VI Effect of Different Guest Molecules

The novel applications of RMs as nanocarriers of biologically active molecules^43^ or as nanoreactors for catalytic reactions^114^ necessitate investigation of the effect of inclusion molecules on the water dynamics of the internal water pools. Furthermore, encapsulation of proteins inside RMs can afford interesting perspectives on their dynamics in confined environments^38,59,115^ and RMs can be used as vehicles to extract proteins from complex mixtures.^36,116^ The current studies both identify initial results and point to important considerations for future guest molecule studies using the ADROSYS methodology.

In particular, such studies provide an opportunity to understand the extent to which guest molecules frustrate the hydrogen-bonding matrix and dynamics of water and to what extent such a frustration varies as a function of confinement. For example, if the inclusion of viscogens reduces the rotational mobility of water in the bulk, one can ask whether or not this effect compounds the slowdown of water in the already motionally restricted environment inside RMs with low *w*_0_, to what extent such a slowdown correlates with a frustration in attempts at hydrogen bonding (as manifest by the chemical shielding), and to what extent the inclusion of viscogens in bulk water mimics or differs from the effect of confining water inside soft nanoenclosures.

#### IV.VI.I Glucose

**Figure 6:**
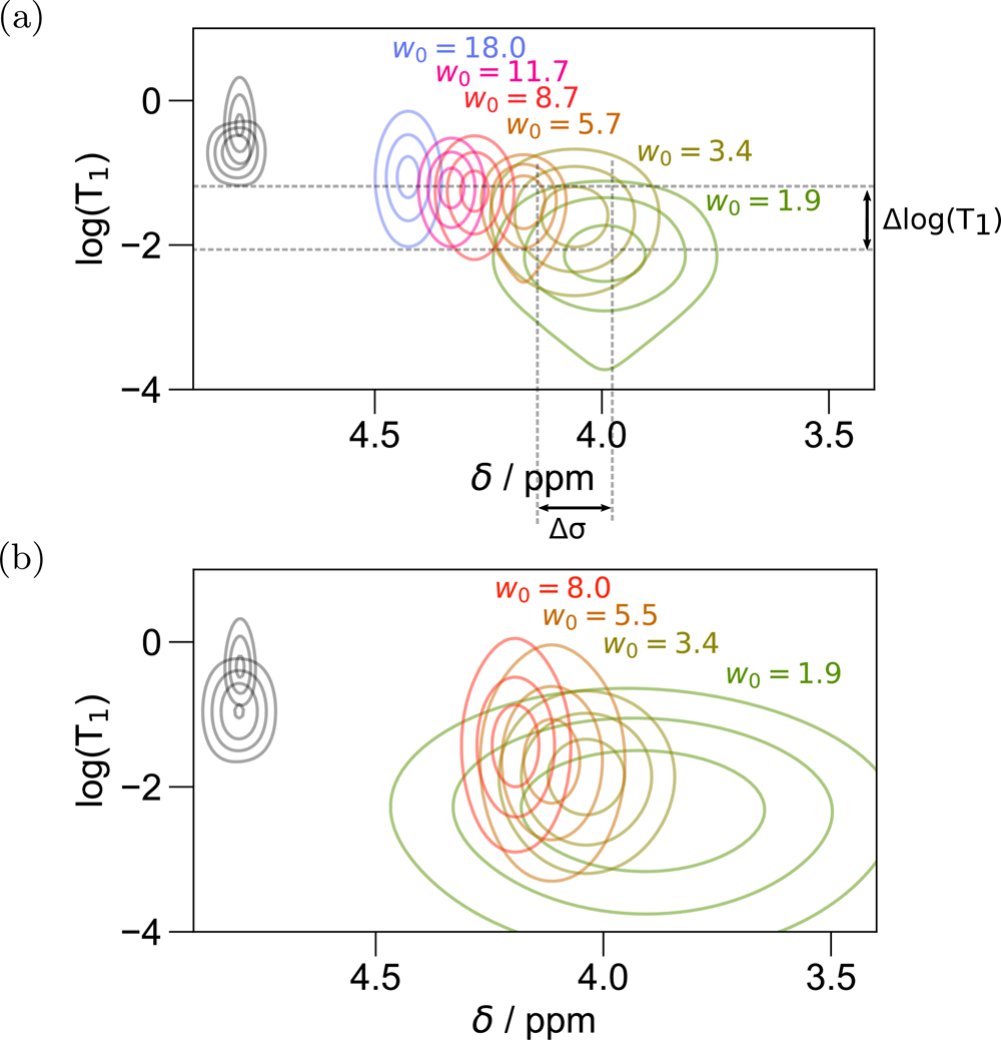
AOT RMs in isooctane prepared using mixtures of guest molecules in D_2_O: (a) 24 wt % glucose and (b) 49 wt % PEG-200. Reported water loadings use the volume of the added mixtures and are therefore similar *w*_0_ RMs are expected to adopt a similar size water pool even though, e.g., the same 49% PEG solution contains about 49% fewer water molecules per surfactant molecule. In both cases, the lower gray set of contours gives the signal for a solution of bulk water with an osmolyte.

Comparing AOT RMs in isooctane prepared with a solution of 24 wt % glucose (Figure 6a) to simple solutions of AOT RMs in isooctane (Figure 3b), there is a noted decrease in *T*_1_, as well as a broadening in line width, especially at low *w*_0_, consistent with a reduction in the rotational mobility of D_2_O. Bulk water measurements containing 20–30% glucose display a viscosity 1.68–2.48× greater than bulk water.^117^ Meanwhile, for RMs above *w*_0_ ≈ 5, eq 5 predicts roughly 2× slower correlation times for the rotational motion of water upon incorporation of the glucose. Therefore, the slowdown in rotational mobility from the addition of viscogens appears roughly additive with the slowdown due to confinement for RMs above *w*_0_ ≈ 5. However, below this threshold, glucose makes a subtle to nonexistent perturbation, implying that interactions with the surfactant dominate. At low water loading, glucose serves mainly to broaden lines along both dimensions (increasing both Δlog(*T*_1_) and Δσ in Figure 6a), an effect that may indicate a greater diversity in the structures of individual RMs, as will be discussed in greater detail with the PEG results.

Comparing similar-sized RMs with glucose (Figure 6a) to AOT RMs without glucose (Figure 3b), the signal coming from high-*w*_0_ RMs with included glucose shifts to more strongly shielded resonances, indicative of a frustration of the hydrogen bonding network that is, again, additive with the effects of confinement by the surfactant. Notably, recent studies have characterized osmolyte-loaded RMs, such as those containing glucose, with high-field NMR,^10^ and explored the impact of the confined environment of these molecules.^118^ In relation to those studies, the experimental tools presented here may help to explain the perturbation to the typical ratio of the β:α anomers of glucose recently observed within an RM.^10^

In general, one must exercise care in the interpretation of ADROSYS results for all guest molecule studies, as two situations might arise. First, as with the hexanol cosurfactant, the guest molecules can contribute additional resonances to the measurement. Second, rapid exchange of deuterons between water and the guest molecule can enable additional *T*_1_ relaxation mechanisms (in addition to rotational motion) that shift the position of the signal along the *y*-axis. Fortunately, both of these effects should contribute relatively weakly, if at all, to the measurements presented here. Briefly (as discussed in Section S5), despite the previously demonstrated slowing of exchange^119^ inside RMs, because of the low mole ratio between the water and the guest molecules, the first situation proves very unlikely. With respect to the second consideration, one can use the bulk solution control measurements (the gray signals in Figure 6a) to provide an upper bound on the extent to which the guest molecule might shift the signal along the relaxation (*y*-) axis.

#### IV.VI.II Poly(ethylene glycol) (PEG)

Provided its molecular weight is sufficiently small (<1 kDa), PEG does not perturb the RM size or geometry.^120^ Typically, the minimal *w*_0_ that can accommodate a given polymer should provide a water pool radius about equal to the hydrodynamic radius of the polymer; failure to meet this condition results in the formation of insoluble precipitates in the solution, otherwise known as “necklacing”.^32,121^ Similarly, here, an inability to form stable RMs above *w*_0_ = 8.0 may be indicative of increasing quantities of PEG destabilizing the RM. Following this caution, PEG as a guest molecule has been shown to lead to interesting results in RM systems. For example, water-soluble polymers such as PEG have been shown to facilitate mixing of the individual water pools,^120,122^ or to impact the bending modulus of the surfactant shell.^123^

Here (Figure 6b), we sought a large weight percentage of PEG in order to ascertain the effects of a high concentration of polymer and to specifically compare to the water inside Igepal RMs; therefore, PEG-200 was incorporated at 49 wt %. The solutions encapsulated in RMs of various sizes in Figure 6b display a decrease in *T*_1_ relative to RMs without guest molecules (Figure 3). Note that the 49 wt % PEG-200 control (bulk solution) shows a shorter *T*_1_ along with a wider distribution along the *T*_1_ and chemical shift dimensions vs bulk D_2_O.

Remembering that Figure 6 (as elsewhere here) labels the samples using the definition of *w*_0_ based on aqueous solution volume (i.e., *w*_0_-equivalent), note that for these 49% PEG data, the water:surfactant molecular ratio is approximately *w*_0_/2, while in Figure 3, the water:surfactant molecular ratio is *w*_0_. Indeed, the RMs containing 49% PEG display *T*_1_ similar to, though still slightly shorter than, “empty” AOT reverse micelles (i.e., without any guest molecules, Figure 3b) of half of the *w*_0_. Thus, they demonstrate a rotational mobility similar to, though still slightly slower than, empty RMs with half the *w*_0_. The chemical shift of the PEG-loaded RMs similarly resembles empty RMs with half the *w*_0_. This result could be related to the finding that PEG tends to associate with the interface of the surfactant,^123^ and might be indicating that such adsorption simply confines the water to a smaller volume in the center of the RM.

Notably, the high concentration of PEG also induces a broadening of the peaks along both dimensions. This broadening may indicate a greater diversity of water microenvironments accompanying differences in the number or conformations of the PEG macromolecules loaded into each RM. Specifically, note that because *T*_2_ > *T*_1_ and the line width must be at least 1/π *T*_2_, a *T*_1_ of 10^–1.5^ s would demand a line width of at least the Δσ displayed in Figure 6a; this explains nearly all of the line broadening of the glucose-containing RMs in Figure 6a, but not that of the PEG in Figure 6b, which likely indicates a true heterogeneity along both chemical shielding and *T*_1_ dimensions, tied to heterogeneity in the aggregate structures. At these concentrations, there is one PEG molecule for every 0.4 kDa of solution. As a reference, compare 0.4 kDa to the fact that eq 2 (combined with 18 g/molecule) predicts that an RM of *w*_0_ = 1.0 will contain only 0.3 kDa of solution, while an RM of *w*_0_ = 2.8 will contain 0.8 kDa. Thus, at very low water loadings, only one (or a handful) of PEG molecules will be included in each RM. This implies a significant variability when constructing RMs, as some will contain a PEG molecule and some will not. The broadening of the 2D peaks observed, especially at lower water loadings, likely arises from the resulting heterogeneity of the RM structure, where some RMs contain PEG molecules, while others do not. This heterogeneity may also indicate that the considerable size of the PEG molecule relative to the RM might be interfering with the typical structure so that even RMs of the same constitution could exhibit different conformations.

#### IV.VI.III Bovine Serum Album (BSA)

To explore the breadth of possible guest molecules, the SI (Section S9) presents data applying the ADROSYS method to solutions of bovine serum albumin (BSA), following protocols from early electron spin resonance (ESR) literature.^124^ Overall, these results are consistent with the known propensity of AOT RMs to unfold proteins but do offer insights toward future studies deploying ADROSYS on protein systems.

### IV.VII Details of Interpretation

From the *T*_1_ values measured in these experiments, approximate values of τ_c_ were determined by using eq S1 and tabulated in Table 1. The quadrupolar coupling constant 230 kHz^22^ was used in all cases. The exact quadrupolar coupling constants in these RM experiments may show some amount of variation given that the quadrupolar coupling constant decreases with an increase in O–H bond length.^125^ Despite the fact that the quadrupolar coupling constants have been determined from MD simulations,^126^ questions arise as to the accuracy of the water model in question^127^ in such determinations. Furthermore, illustrated by Figure S2, correlation times over a few hundred nanoseconds will actually exhibit a relaxation time somewhat shorter than the relaxation indicated by the motional narrowing limit approximation (eq 5). Therefore, Table 1 simply approximates for all cases using the quadrupolar coupling constant for bulk D_2_O. Accurate determination of the quadrupolar coupling constants, and accounting for deviations from eq 5 at longer values of τ_c_, would yield greater accuracy in the determination of the values of τ_c_ (Table 1) in these confined water environments. Such potential studies represent important future directions. The current investigation focuses less on the detailed refinement of the models used to interpret the data and chooses to accept a simplified interpretation. It can then proceed to interrogate stark and qualitative (and still valid) trends in how the signal migrates through the 2D correlation plot as the size of the confinement changes.

**Table 1:** Values for τ_c_ Determined from the *T*_1_ Measurements for Various RM Samples

surfactant	solute/guest	dispersant	*w*_0_	τ_c_ (ps)
AOT		isooctane	0.9	1276.9
AOT		isooctane	3.5	80.6
AOT		isooctane	6	16.1
AOT		isooctane	9.3	12.8
AOT		isooctane	12.8	9.0
AOT		isooctane	20.7	8.1
AOT		hexane	0.9	805.7
AOT		hexane	1.8	80.6
AOT		hexane	2.3	57.0
AOT		hexane	3.3	22.7
AOT		hexane	5.7	16.1
AOT		hexane	8.9	10.1
AOT		hexane	12.3	9.0
AOT		hexane	20.3	6.4
AOT		CCl_4_	0.9	805.7
AOT		CCl_4_	1.6	160.8
AOT		CCl_4_	2.7	71.8
AOT		CCl_4_	5.2	20.2
AOT		CCl_4_	7.4	12.8
Igepal		cyclohexane	1	25.5
Igepal		cyclohexane	1.8	22.7
Igepal		cyclohexane	3.3	10.1
Igepal		cyclohexane	5.4	9.0
Igepal		cyclohexane	7.8	8.1
Igepal		cyclohexane	14.7	5.7
CTAB^a^		hexane	4	25.5
CTAB^a^		hexane	9	22.7
CTAB^a^		hexane	12.7	10.1
CTAB^a^		hexane	15.6	9.0
CTAB^a^		hexane	20.1	8.1
AOT	glucose^b^	isooctane	1.9	160.8
AOT	glucose^b^	isooctane	3.4	80.6
AOT	glucose^b^	isooctane	5.7	32.1
AOT	glucose^b^	isooctane	8.7	20.2
AOT	glucose^b^	isooctane	11.7	12.8
AOT	glucose^b^	isooctane	18	10.1
AOT	PEG-200^c^	isooctane	1.9	202.4
AOT	PEG-200^c^	isooctane	3.4	101.4
AOT	PEG-200^c^	isooctane	5.5	80.6
AOT	PEG-200^c^	isooctane	8	25.5

a Hexanol as cosurfactant.

b 24%-weight glucose as guest molecule.

c 49%-weight PEG-200 as guest molecule.

**Figure 7:**
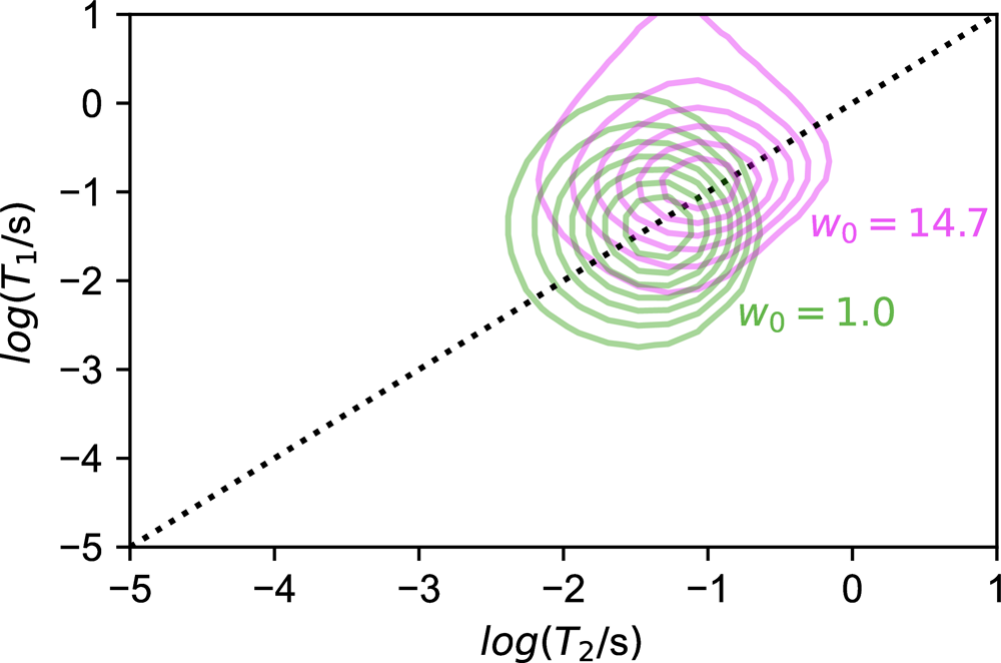
Results for D_2_O correlated *T*_1_–*T*_2_ relaxation measurements for the lowest and highest water loadings of Igepal/cyclohexane reverse micelle samples. A dotted line depicts values for which *T*_2_ = *T*_1_.

A full 2D relaxation-relaxation correlation (i.e., 2D relaxometry) experiment can test whether a particular population of D_2_O exhibits relaxation in the motional narrowing regime. Specifically, as noted in the text after eq 5, *R*_2_ = *R*_1_, i.e., *T*_1_ = *T*_2_, if and only if the correlation time falls in the motional narrowing regime (i.e., much faster than the resonance frequency). Note that such a 2D relaxometry experiment (generated from relaxation decays in both dimensions) differs from the 1.5D relaxometry experiments throughout the rest of this paper, where relaxation decay supplies one dimension (the *T*_1_), while a Fourier transform of resonant oscillations (i.e., standard chemical shift) supplies the other dimension of the correlation. In a 2D relaxometry experiment, a stroboscopic experiment replaces the standard direct detection dimension (*t*_2_). As an example, we tested whether or not the water inside Igepal remains in the motional narrowing regime for all values of *w*_0_. In Figure 7, correlated *T*_1_ – *T*_2_ measurements were carried out for Igepal reverse micelles for small and large *w*_0_: *w*_0_ = 1 and *w*_0_ = 14.7, respectively. In both cases, the *T*_1_ – *T*_2_ distribution is unimodal and approximately centered about the *T*_1_ = *T*_2_ line, indicating that D_2_O is in the extreme narrowing limit. Small off-centering of the signal or small peaks in other part of the correlation map may be considered artifactual.^128^

## V Conclusions

The correlated change in water chemical shift and *T*_1_, in general, tells a consistent story indicating that confinement leads to a dramatically nonbulk-like environment when water is confined inside small RMs, where the hydrogen bonding matrix of the water pool is frustrated and where the water rotational dynamics are slowed. Here, automated measurements of water confined under a range of conditions probe the 2D correlation between relaxation and the chemical shift. These measurements observe changes spanning a few orders of magnitude of relaxation times and chemical shift variations of ∼25%. Water, of course, comprises a dynamic structure of fluctuating hydrogen bonds, constantly swapped and rearranged as the water undergoes not only rotational but also translational diffusion. Interestingly, these results here observe an inflection point in the shape swept out by the correlated measurements near *w*_0_ = 3 to 5 (*w*_0_ ≡ [H_2_O]/[surfactant]) with a fair degree of consistency. The notable exception to this result was for the Igepal surfactant, which likely forms a structure with polyethoxylate groups oriented inward, surrounded by, and hydrogen bonded to, the water. Note that for RMs of 3 < *w*_0_ < 5, eq 2 expects somewhere between 70 and almost 200 water molecules inside the RM so that an apparent breakdown in the dynamic structure of water inside RMs of this size proves quite interesting. For water confined inside Igepal RMs, while changes in diamagnetic shielding indicating a change in average hydrogen bonding strength remain prominent, it is not correlated with as prominent a retardation of the rotational diffusion so that the collection of signals in the resulting ADROSYS spectrum trace out a fundamentally different shape.

Here, ADROSYS was demonstrated in RMs, as they provide systems where the levels of confinement can be easily adjusted and where response of the water matrix to the incorporation of guest molecules can be easily interrogated. However, this technique is ready both for wide-scale application to various confined water systems and for integration with standard measurement suites. Future applications of this deuterium NMR screening technique might span, for instance, samples ranging from structured porous silicates to membraneless organelles or other liquid–liquid phase separation systems of biological relevance.

This publication focused on the deployment of an automated technique, thus providing a method to screen or survey differences in confined water pockets, and through this focus was able to demonstrate striking qualitative differences between different RM systems. With regard to further investigation of the nuances of how to best interpret changes in *T*_1_ and chemical shift under such extreme circumstances, it presents the possibility of further deploying these measurements, alongside the *T*_1_ vs *T*_2_ test demonstrated here, to more exactly quantify the correlation time vs quadrupolar coupling constants for the smaller pockets of confined water that were studied here. Standard models (Figure S2) indicate that changes to relaxation rates, and evidence of departure from the motional narrowing regime, should be observable for the smallest *w*_0_ AOT reverse micelles that were observed here. This departure should enable subsequent studies to experimentally tweeze apart the correlation time from any variations in the quadrupolar coupling constant and would offer insight into the exact time scales of diffusion inside these smallest reverse micelles, as well as the impact of the electric field gradients imposed by ionic side chains under various extreme confinements. When considering protein guest molecules, a clear next step would involve exploring choices of surfactant^40,129^ that enable encapsulation of folded proteins in order to study the hydration water of such proteins. In particular, such studies could explore the utility of freezing the encapsulated proteins in order to “shed” the water^115^ or of the “evaporation-injection” method,^40^ both of which offer the promise of encapsulating large macromolecules with this method inside smaller (lower *w*_0_) RMs, thus increasing the ratio of the protein solvent-accessible surface area (SASA) to the RM water molecules (without inducing transitions in the protein system). Finally, these studies set the stage for an analogous solution-state ^17^O measurement. The oxygen nucleus does not exchange with guest molecules, and in the past,^130^^17^O has identified the presence or absence of the impact of exchange effects in deuterium relaxation spectroscopy.

## Supporting Information Available

Meta-analysis and fitting of the number of molecules per RM, fitting of the core–shell model, and further details and data pertaining to considerations regarding proton exchange (PDF)
Details of the automation program and accompanying processing (PDF)
