# Supplementary material for: Rapidly Screening the Correlation between the Rotational Mobility and the Hydrogen Bonding Strength of Confined Water
Source: J Phys Chem B. 2024 Oct 23;128(43):10749–63. doi: 10.1021/acs.jpcb.4c05397 (PMC11533181; doi:10.1021/acs.jpcb.4c05397)
Supplement: Supplementary file 1 — jp4c05397_si_001.pdf [file jp4c05397_si_001.pdf]

Supplementary Information for: Rapidly Screening the Correlation Between the Rotational Mobility and the Hydrogen Bonding Strength of Confined Water

Alec A. Beaton,[1] Alexandria Guinness,[1] and John M. Franck[1]
[1]Department of Chemistry, Syracuse University, Syracuse, NY 13210, USA*
(Dated: Saturday 10th August, 2024)

S1. Meta-Analysis and Fitting of $\bar{n}$

As indicated in the text, we performed a meta-analysis where we digitized data from a series of previous publications pertaining to $\bar{n}$ (the aggregation number, or number of surfactant molecules per individual reverse micelle). Fig. S1 shows the data digitized from previous publications and fits to the form indicated by Eq. (2) (main text). The points are chosen for the least squares fit following the reasoning that vapor-pressure osmometry data serves as a better guide at lower water loading, while data that relies on centrifugation and other techniques assuming a constant density serve as better guides at higher water loading. As indicated in[S1], the osmometry data is expected to have less value for higher water loading, explaining the one significant outlier from the fit here. See the main text for more details.

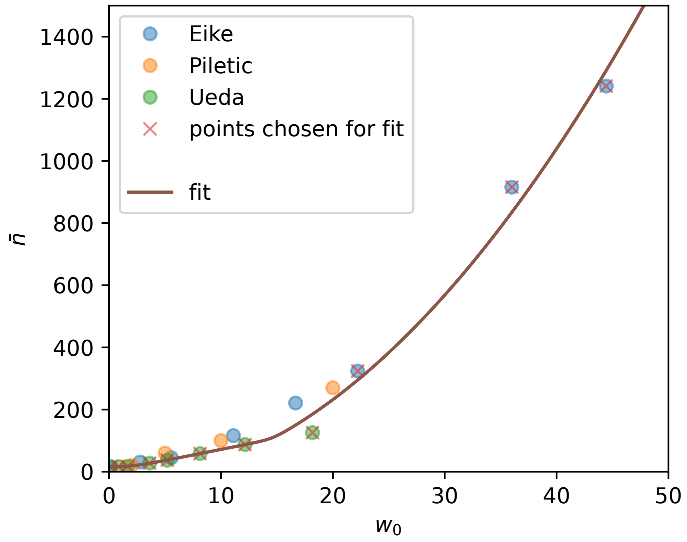

FIG. S1. Fitting of a consensus number of water molecules as a function of reverse micelle (RM) water loading. Data digitized from[S1–S3] is shown in the legend, as indicated by first author.

S2. Background on Quadrupolar Relaxation

Quadrupolar relaxation, for deuterium ($I = 1$), is given by[S4]

$$R_1 = \frac{3}{80}\left(\frac{e^2Qq}{\hbar}\right)^2 \left[J(\nu_D) + 4J(2\nu_D)\right] \tag{S1}$$

where e is the elementary charge, Q is the quadrupolar moment of the nucleus, q represents the electric field gradient at the nucleus, ν_D is the deuterium resonance frequency, and $J(\nu)$ is the reduced spectral density function (the Fourier transform of a normalized auto-correlation function that describes the rotational motion).[S5] Note that, for clarity, J here accepts an argument with units of Hz (as resonance frequencies are typically given in Hz), so that the form of $J(\nu)$ here is given by:

$$J(\nu) = \frac{\tau_c}{1 + (2\pi\nu\tau_c)^2} \tag{S2}$$

(In other references, one might find J with an argument of ω with units of rad/s that yields an equivalent value for $\omega = 2\pi\nu$.) Similarly, the rate of transverse relaxation, R_2, is given by,

$$R_2 = \frac{1}{160}\left(\frac{e^2Qq}{\hbar}\right)^2 \left[9J(0) + 15J(\nu_D) + 6J(2\nu_D)\right] \tag{S3}$$

S3. Test of Core-Shell Model

The model of Fig. S3 assumes weighted core and shell contributions to the T_1 time and to the chemical shift. The values shown here interpolate between a chemical shift of 3.82 for shell water and 4.72 for core water, and between $\log(T_1/\mathrm{s})$ of -2.64 for shell water and -0.89 for core water. The thickness of the shell water that yields this fit is 0.15 nm, notably less that a layer of water, and likely also arguing against the validity of a core-shell model. Also, as noted in the main text, while averaging of relaxation rates ($T_1^{-1} \propto \tau_c$) of core *vs.* shell water molecules represents a physically reasonable situation, averaging of the relaxation times ($T_1 \propto \tau_c^{-1}$) does not.

S4. Susceptibility

The susceptibility of different RM solutions varies. On the same day, the relative susceptibility is given by the relative frequencies of the TMS resonance. The absolute susceptibility can be determined by looking at the experiments where a capillary of water with sodium trimethylsilylpropanesulfonate (DSS) was added into the sample.

* jmfranck@syr.edu

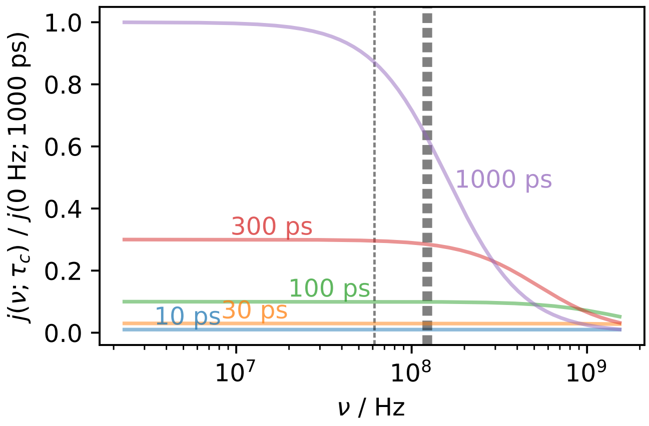

FIG. S2. Displays the standard spectral density function, and how it varies with correlation time (different colors). (Curves shown here correspond to an exponential rotational correlation function.) The longitudinal relaxation rate ($R_1 = 1/T_1$) samples the spectral density at the deuterium resonance frequency ($\nu = \omega_D/2\pi$, marked by the thin dashed gray line) and at $2\nu = \omega_D/\pi$ (marked by the thick dashed gray line – this value is scaled by 4), giving rise to Eq. (S1). The initial flat portion of each curve corresponds to the motional narrowing regime, and if the relaxation rate samples the spectral density curve in this regime, then Eq. (5) (main text) approximates the relaxation rate very well. As the rotational correlation time (indicated above each curve) increases, the spectral density of interactions arising from rotational motion spreads out in the frequency domain, leading to less relaxation in the motional narrowing regime. The values of ν_{rf} and $2\nu_{rf}$ marked here correspond to a magnetic field of 9.4 T (9.4 T$\gamma_H/2\pi = 400$ MHz).

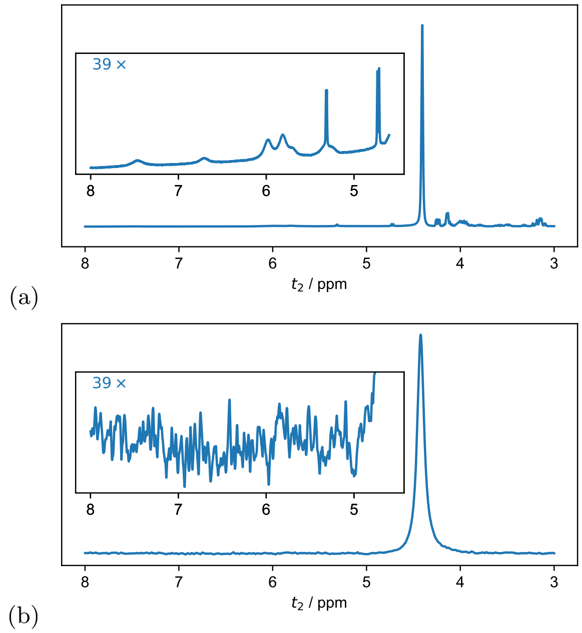

FIG. S4. Spectra of water resonance and the surrounding region. In both spectra, the largest peak shown comes from the (heavy) water resonance. In the proton spectrum (a), the region downfield from the water clearly displays the exchangeable protons from glucose (see[S6]). In the deuterium spectrum (b), any such resonances are either buried in the noise or significantly broadened. (The proton spectrum is referenced to TMS at 0 ppm and the deuterium spectrum is referenced to the upfield hexane peak at 0.819 ppm. Both spectra show processed data from TopSpin, with windowed Fourier Transform and automated baseline correction implemented *via* `efp;apk0;apk;abs`).

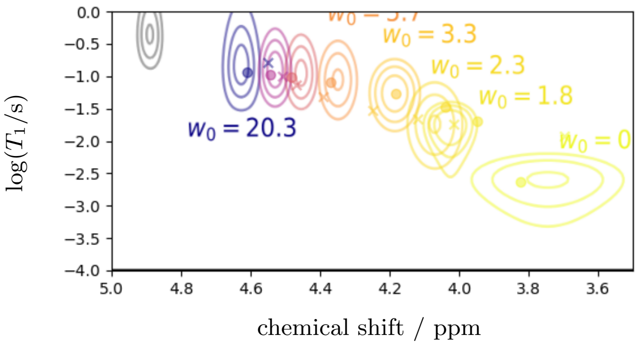

FIG. S3. Filled circles, "x" markers, and contours are all colored according to water loading w_0 (utilizing an earlier color scheme than the perceptually uniform one in the main text.) The "x" markers illustrate the best-fit attempt to fit a single core/shell model to the center of the distributions – where the relaxation rates (*equiv* correlation times) average between the core and shell. The filled circles illustrate a similar best-fit attempt when the values of the relaxation time T_1 (*equiv.* $1/\tau_c$) are assumed to vary linearly with the fraction of core *vs.* shell. Both cases look for a linear averaging of chemical shielding/shift between core and shell.

The susceptibility of water is known, allowing us to calculate the exact field, and then the TMS frequency of the RM solution gives the susceptibility of the RM solution.

S5. A note on proton exchange in glucose

Glucose, like other solutes, when encapsulated in reverse micelles (RMs) experiences a decrease in the rate of proton exchange (relative to the rates in bulk aqueous solution) that exhibits a dependence on w_0.[S6] Initiation of proton exchange is a rare event, but once initiated, it can propagate via standard proton exchange mechanism (*e.g.*, Grotthuss). Assuming that such mechanisms cannot propagate through the aprotic solvent, it's expected that exchange should be dramatically reduced in reverse micelles for all cases. Therefore, the rates of proton exchange are expected to be slower than the relaxation rates observed here and, therefore, not to have a dramatic affect on the present measurements.

The timescale for exchange of water protons onto glucose inside RMs have been shown to be on the order of tens of ms[S6] (with pseudo-first order rate constants in the tens of s^{-1}). In the current paper, the glucose concentration is approximately 1.4 M (similar to, though slightly more than[S6]), so that there are approximately 40× less

glucose molecules *vs.* water. The ^{1}H spectrum of glucose inside RMs with (proton) water (Fig. S4a), show both that (consistent with[S6]), the exchangeable protons on glucose exchange slowly enough that they can be resolved from the protons on the water, and that these resonances are much smaller than the resonance from the water proton. By comparing this spectrum (Fig. S4a) with the ^{2}H spectra inside RMs with D_2O (Fig. S4b), one can specifically see that, even with 128 signal averages, ^{2}H resonances with similar chemical shifts cannot be detected. (This, notably, contrasts with the studies involving hexanol surfactant in Fig. 5b (main text), where – especially at low water loading where the -OD resonance is more prominent – the ratio of moles of hexanol to moles of water is 1:1 or even higher.) Thus, it is highly unlikely that the glucose resonances themselves contribute in any significant way to the data presented in the guest molecule studies.

The exchange of deuterated water with glucose could significantly affect the apparent relaxation rate of the deuterated water. In particular, the slow rotational correlation time of glucose (expected to be significantly slower than that of water) could lead to very fast relaxation of the deuterium spins once they exchange onto the glucose. In this case, the effect on the apparent relaxation rate of the deuterated water would scale with the ratio between the exchange rate (units of M/s) and the total moles of deuterons (units of M). The low ratio of glucose molecules to water molecules (1.4 M *vs.* 55.4 M), means that exchange with any of the glucose sites will alter the (deuterated) water Nuclear Magnetic Resonance (NMR) signal 40× less than it alters the glucose signal; but we must consider that such an effect may not be completely negligible. Dramatically increasing the relaxation rate of the deuterium spins on glucose could effectively suppress the ^{2}H NMR signal from the glucose sites. Because there are 40× more water molecules *vs.* glucose molecules, fast quadrupolar relaxation of the exchangeable sites on glucose (or, similarly, PEG) can suppress the glucose resonances without dramatic changes to the relaxation of the water. Fortunately, one can place an upper bound on such an effect, by noting that the fastest exchange – and therefore most dramatic effect – should occur for glucose dissolved in bulk D_2O, so that the differences between the two grey resonances in Fig. 6a (main text) should exceed the upper bounds on the change to the relaxation time that might arise from exchange. Note that the difference in the relaxation times indicated by the two grey peaks in Fig. 6a (main text) must actually exceed the change that could be induced by such an exchange effect, since at least some part of the difference in relaxation times must come from the fact that the D_2O molecules rotate more slowly in the viscous glucose solution. As noted previously[S6], this exchange is small and becomes smaller as the size of the RMs (*via* w_0) becomes smaller.

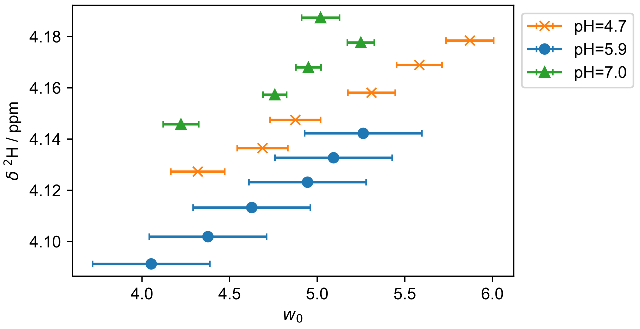

FIG. S5. The ^{2}H chemical shift (and the ^{1}H chemical shift, not shown) depend slightly on pH. However, the change in chemical shift with w_0 tends to be larger. Here, lyophilized AOT was titrated with 50:50 H_2O:D_2O to yield a series of samples with increasing w_0, allowing for a controlled simultaneous measurement of chemical shift and water loading. The error bars give the standard deviation between three different means for determining the water loading from the ^{1}H NMR spectrum.

S6. pH dependence

As noted in previous literature[S6], alterations to the pH inside the RM can lead to changes to the proton exchange times. For that purpose, note that the pH of the AOT shown for these measurements, as well as the 2D relaxometry measurements in the main text, was as supplied from the vendor and measured to be a pH of 5.9 in 1 mg/L solution ([S7]).

Furthermore, the pH might perturb the current measurements, a possibility especially likely for low w_0 Aerosol-OT (AOT), where the buffering capacity of AOT likely controls the pH.

Titration of a 1 mg/mL aqueous solution of AOT, followed by repeated lyophilization and re-dissolution enables one to prepare an AOT solution with a particular target value[S7]. Implementation of this procedure in the authors' lab led to the additional observations that (1) a freeze thaw cycle help to equilibrate the pH of the mixture, making many lyophilization cycles unnecessary and (2) a 1 mg/mL concentration resulted in a large lyophilization volume for the samples required here, so some samples were prepared with a 2 mg/mL aqueous solution. Preliminary measurements (not shown) showed insignificant changes in T_1, so we focused on analyzing the change in chemical shift. These measurements focused in particular on low water loading, where the interactions between the water and the head-group of the surfactants likely dominate over other effects. Specifically, to a solution with approximately 47 mM AOT (as well as small amounts of hexane and TMS) was added 940 mol (8.58 µL) of 50:50 H_2O:D_2O per liter of solution, yielding a RM solution with $w_0 = 20$. NMR tubes were loaded with a different, "dry" solution (lyophilized or, in the case of unadjusted AOT, AOT from the vendor stored in a desiccator) of 300 mM AOT in isooctane.

An initial 940 mol aliquot of 50:50 H_2O:D_2O per liter of solution was pipetted directly into the NMR tube and capped with aluminum foil and teflon tape. The NMR tube was then inverted three times and vigorously shaken by hand and let to sit for 10 minutes prior to acquiring an initial 1D ^{1}H and ^{2}H NMR spectra. Both 1D ^{1}H and ^{2}H NMR spectra were measured after each subsequent addition of an aliquot of the $w_0 = 20$ solution, with aliquot sizes ranging between 40 and 45.2 µL (calculated in advance to yield an even spacing of w_0). Because of the small w_0 probed, and the effect of w_0 on the chemical shift, the exact w_0 was measured by NMR integration of the ^{1}H spectrum. Due to the complex peak pattern of the AOT frequently overlapping with the water, and the presence of some amount of water even in "dry" AOT, 3 approaches quantified the amount of water: Noting that the position of the water peak changes with water loading, the integrals of three regions were calculated: "A" from 3.05-3.34 ppm – representing 2 AOT protons; "B" from 3.89-4.47 ppm – representing a region that for very low w_0 ("dry") samples contains only peaks from 5 AOT protons; and "C" from 3.64-4.47 ppm – representing a region that includes *both* 5 aot protons (as in "B") but also water. In addition to the spectra of interest a "water-free composite" spectrum was also constructed by manually piecing together signals from different water loadings to construct a spectrum showing only AOT spectrum, with no water contribution. In all cases, the spectra were normalized to set the integral of the "A" peak to 2. Noting that the "dry" spectrum contained two protons for every water molecule, while the aliquoted H:D water mixture contained only 1 proton per molecule, the w_0 value was then determined according to the following 3 values: (1) 0.5× the "C" integral and 0.5× the "B" of the normalized "dry" spectrum were subtracted from the "C" integral of the normalized spectrum of interest (2) 0.5× the "C" integral of the normalized "dry" spectrum and the value 7.5 (5 AOT protons in the spectrum of interest, and 0.5×5 to account for AOT protons in the "dry" spectrum) were subtracted from the "C" integral of the normalized spectrum of interest and (3) the integral of the "B" of the water-free composite spectrum, 0.5× the "C" integral of the normalized "dry" spectrum, and 0.5× the "B" integral of the normalized "dry" spectrum, were subtracted from the "C" integral of the normalized spectrum of interest. (The highest chemical shift datapoint in Fig. S5 is believed to be an outlier representing an error, and the value of w_0 should be higher based on the amount of water added, but it is shown for completeness.)

S7. Relation to Other Types of Measurements

Note that studies of RMs by Dynamic Light Scattering (DLS) have been performed in the past, though have not been repeated here, because we expect the polydispersity measurements by DLS to be similar or weaker reliability compared to the T_1 distributions presented here, since both rely on analysis of multivariate exponential decays. Furthermore, DLS is generally considered optimal for larger sized particles and has shown significant discrepancy with the measurements of particle diffusion garnered from NMR diffusion measurements, which are expected to be quite accurate and more optimal for smaller particle sizes. It is worth noting that significant groundwork has also been laid to relate the present types of measurements to molecular dynamics simulations.[S8]

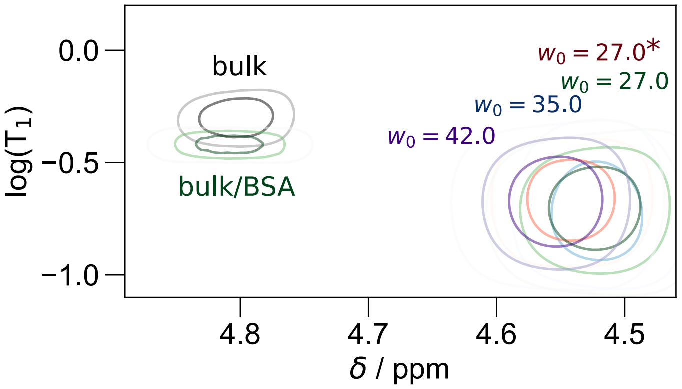

FIG. S6. Bulk D_2O shown in gray with a bulk D_2O/BSA solution shown in green. At lower chemical shifts are a range of reverse micelles at higher water loadings (w_0 = 27, 35, 42) containing BSA, demonstrating that two T_1 distributions are not observed. An RM without BSA of $w_0 = 27$ (indicated with asterisk) is also shown, demonstrating that RM-encapsulated BSA has negligible impact on chemical shift or T_1. (Because of the variety and high water loading of samples here, the color scheme doesn't adhere to that of the other figures in the text.)

S8. Low Weight Percent PEG-200

Comparing AOT RMs in isooctane prepared with a solution of 1 w% PEG-200 to simple solutions of AOT RMs in isooctane, RMs containing PEG-200 exhibit slightly shorter T_1 as those without. This indicates a slight reduction in the rotational mobility in the presence of the PEG solution which is expected given the small quantity of PEG added to the solution. Additionally the chemical shifts in the presence of PEG are shifted to slightly lower frequencies. This is indicative of the D_2O engaging in less hydrogen bonding to the water matrix, which is again expected as it would be engaging with the PEG molecule. The linewidths with PEG inclusion are slightly larger than those without, which is consistent with the observed slight decrease in T_1.

S9. Bovine Serum Album (BSA)

Bovine serum albumin (BSA) is a widely available and commonly studied protein. Often used to mimic the effects of molecular crowding in the cell, BSA offered here a unique opportunity to study how encapsulation of a protein might affect the confined water within the RM. Previous studies that tracked changes to 2D NMRs (HSQC) spectra of ubiquitin as a measure of the level and character of macromolecular crowding reported that BSA interfaces (in crowded solution) and RM interfaces

(*i.e.*, encapsulating the ubiquitin) both yielded a similar crowding effect.[S9] This leads to the base expectation that in RMs with BSA guest molecules, the surfactant and BSA surfaces will yield an additive effect when confining the water. A related serum albumin protein, Human Serum Albumin (HSA), was incorporated into AOT RMs and the environment studied by ESR spectroscopy as a function of w_0 and was found to restrict the rotational diffusion of the protein at smaller w_0[S10]; this observation also points to an additive retardation of diffusion when comparing HSA in aqueous solution *vs.* inside the RM. However, as indicated in other literature[S11,S12], surfactants (especially the AOT employed here) frequently interact with and disrupt the native conformations of proteins, and careful choices of surfactant are required to maintain the native fold.

Samples for BSA followed the general protocol outlined here for guest molecule studies. For the BSA samples, the stock solution contained 50 mM Tris (buffer, Thermo Fisher Scientific) in D_2O and the pH was adjusted to 7.4, following established preparation procedure[S10], to which 0.02 mM BSA (Sigma Aldrich) was added.

Here, the impact of 600 µM (4 wt/v%) BSA on the properties of the internal water pool was investigated. Note that at this concentration, there is approximately 1 BSA molecule per every $\sim$ 1700 kDa of solution; while the sizes of the water pools for $w_0 = 27$, 35, and 42 are $\sim$ 220 kDa, $\sim$ 500 kDa, and $\sim$ 870 kDa (Eq. (2) (main text)). Therefore, we isolate this study to higher w_0 values compared to the other studies in this paper, since moving to lower w_0 would result in solutions where the number of RM aggregates far exceeded the number of BSA molecules; already, from the estimated sizes above, $w_0 = 27$, 35, and 42 correspond to approximately 7.6, 3.4, and 1.9 RM per BSA molecule.

As 4% seems to be a small concentration, for a sense of scale, it is worth considering two spheres, equal in density, that differ in weight by 100% to 4% – *i.e.* the most simplistic model for BSA molecules loaded one per RM. Under these conditions, the radius of the smaller sphere would be $\sim$ 34% of the larger sphere, while the surface area would be $\sim$ 12%. To form a slightly more accurate estimate of the relative surface areas of RM *vs.* BSA in these samples, note that at $w_0 = 42$, Eq. (1) (main text) indicates the surface area of the surfactant inside the reverse micelle will be 678 nm^2. At $w_0 = 27$, it predicts a surface are of 280 nm^2. Meanwhile, BSA presents a solvent-accessible surface area (SASA) of about 300 nm^2 (notably larger than the spherical prediction above, due to surface roughness) when folded – a value that may become greater if the BSA unfolded without migrating to the surfactant interface. Thus, differing methods of estimation predict that loading BSA into a RM increases the surface area of the water by anywhere from 12% to 93% of the surface area in the absence of BSA. Based on a strictly core-shell argument, and following on the previous observations that frustration of the water matrix due to confinement *vs.* inclusion of guest molecules seemed to be additive, one would expect water inside BSA-loaded reverse micelles to behave somewhat differently as compared to unloaded micelles.

As seen in Fig. S6, the presence of BSA only subtly impacts the properties of the internal water pool observed by this measurement, with a very slight shift to slower rotational mobility (shorter T_1) and more frustrated hydrogen bonding (lower chemical shift) *vs.* the "empty" D_2O RMs. Specifically focusing on $w_0 = 27$, where one might expect a BSA molecule loaded into roughly 50% of the RMs, significant additional separation or broadening of the peak along the T_1 and/or chemical shift dimensions does not occur, even though both broadening and 2 separate 2D peaks have been observed in other measurements reported here. Notably, even at this low concentration, a core/shell model would lead one to believe this geometry leads to a significant increase in more "hydration"-like water (*i.e.* an "inner shell") that would result in noticeable changes to chemical shielding and T_1 – however, if such an increase is present, it is extremely subtle. A likely interpretation for this minimal change could be that the BSA unfolds and interacts with the AOT, as observed in previous studies, thus failing to dramatically alter the surface area of the water pool. Subsequent comparitive studies involving other surfactant systems, such as cetrimonium bromide (CTAB) and/or 10MAG/LDAO, with this methodology may offer insight into how the interactions involving different lipid systems and the hydration water might play a role in stabilizing the native structure of proteins inside the RM.

S10. Automation and Pulse Program Scripts

A. Automation Program

The automation program ("AU" program) is as follows:

```
FILE *logfp;
char logpath[1000];
char titlestr[1000];
double peakFreqHz, peakFreqPPM, peakIntensity, maxpsh,
    maxpsp, maxips, first_maxpsp;
double sf, sfo1, o1;
float p1;
// p1_list should be length-3 array for pulse
// lengths around 180 time
// nutation_peaks should be length-3 array with
// peak intensities (sign matters)
// bit for linear regression
// pull y-intercept/2 for p1
float p1_list[3] = {660.0, 720.0, 780.0};
double nutation_peaks[3];
int noofscans, pscal_save, i, j, numPeaks;
int ns;
float repDelay;
float my_d1;
int stillgoing;
#define REAL double
#define MIN_DOUBLE 1e-9

REAL p1_list_forreg[3];
// the following are just function definitions,
// with the code given at the bottom, after the quit
// statement
int linreg(int, const REAL *, const REAL *, REAL *,
```

```
           REAL *, REAL *);
void settitle(char *);
stillgoing = 1;

//{{{ open log file
GETCURDATA; // pulls the info about the foreground
            // dataset
(void)sprintf(logpath, "%s/%s/%d/logfile", disk, name,
              expno);
if ((logfp = fopen(logpath, "wt")) == NULL) {
  Proc_err(DEF_ERR_OPT, "Can't open %s\n%s", logpath,
           strerror(errno));
  return 0;
}
fprintf(logfp,
        "hello! I am a log file, and I live in expno %d "
        "-- new version\n",
        expno);
//}}}

//{{{ 1H experiment
RPAR("ab_1H_zg", "all");
fprintf(logfp,
        "Beginning 1H NMR 1D experiment in %d...\n",
        expno);
RPROCNO(1); // sets procno
SETCURDATA  // pull the information for a particular
           // dataset -- in contrast,
           //              getcurdata pulls the foreground
           //              dataset
               settitle("NMR experiment w/ standard "
                        "parameters");
RGA ZG_OVERWRITE
    // ERRORABORT returns from AU or AU subroutine
    //             with value of AUERR (if it is less than
    //             0) there are a couple diff options
    //             (table 2.22 in AU programming manual)
        ERRORABORT;
EF; // FFT w/ exponential apodization
ERRORABORT;
APK; // auto phase (0 and 1)
fprintf(logfp, "Finished 1H NMR 1D experiment.\n");
//}}}

//{{{ 2H experiment for peak picking
IEXPNO;
SETCURDATA; // see comment below about IEXPNO
sprintf(titlestr, "2H to find resonance");
settitle(titlestr);
RPAR("ab_2H_zg", "all");
fprintf(logfp,
        "Beginning 2H NMR 1D experiment for peak "
        "picking in %d...\n",
        expno);
sf = 0.0;
sfo1 = 0.0;
o1 = 0.0;
p1 = 0.0;
//{{{ pull all info
REXPNO(expno);
RPROCNO(procno);
SETCURDATA
//}}}
FETCHPAR("SFO1", &sfo1);
FETCHPAR("O1", &o1);
fprintf(logfp, "here is sfo1: %f\n", sfo1);
fprintf(logfp, "here is o1: %f\n", o1);
// pull + set number of scans
FETCHPAR("NS", &noofscans);
fprintf(logfp, "I got %d scans\n", noofscans);
ns = 1;
STOREPAR("NS", ns);
FETCHPAR("NS", &noofscans);
fprintf(logfp, "after change, I got %d scans\n",
        noofscans);
// pull + set r.d.
FETCHPAR("D1", &repDelay);
fprintf(logfp, "I got d1: %f\n", repDelay);
my_d1 = 2.5;
STOREPAR("D1", my_d1);
FETCHPAR("D1", &repDelay);
fprintf(logfp, "after change, I got d1: %f\n", repDelay);
RGA;
ZG_OVERWRITE;
ERRORABORT;
EF;
ERRORABORT;
APK;
// PSCAL appears to be for vertical scaling. We want to
// set
//        it to "global."
FETCHPAR("PSCAL", &pscal_save);
STOREPAR("PSCAL", 6);
fprintf(logfp, "pscal_save is %d\n", pscal_save);
PP; // this tells it to run peak picking
ERRORABORT;
numPeaks = readPeakList(PROCPATH(0));
fprintf(logfp, "I find %d peaks\n", numPeaks);
// {{{ store the max peak intensity, frequency, and ppm
// in maxip[s,h,p]
maxips = 0.0;
maxpsh = 0.0;
for (i = 0; i < numPeaks; i++) {
  peakIntensity = getPeakIntensity(i);
  peakFreqHz = getPeakFreqHz(i);
  peakFreqPPM = getPeakFreqPPM(i);
  if (peakIntensity > maxips) {
    maxips = peakIntensity;
    maxpsh = peakFreqHz;
    maxpsp = peakFreqPPM;
    maxpsp = peakFreqPPM;
  }
}
freePeakList();
// }}}
FETCHPAR("SF", &sf);
fprintf(logfp, "I got %f for SF\n", sf);
sfo1 = sf + maxpsh * 1.0e-6;
fprintf(logfp, "I got %f for SFO1 to set\n", sfo1);
STOREPAR("SFO1", sfo1);
FETCHPAR("SFO1", &sfo1);
FETCHPAR("O1", &o1);
FETCHPAR("P1", &p1);
fprintf(logfp, "I set SFO1 to %f\n", sfo1);
fprintf(logfp, "I set O1 to %f\n", o1);
fprintf(logfp, "I set SF to %f\n", sf);
fprintf(
    logfp,
    "I get P1 of %f (which comes from the par file)\n",
    p1);
// pull + set number of scans
FETCHPAR("NS", &noofscans);
fprintf(logfp, "I got %d scans\n", noofscans);
ns = 1;
STOREPAR("NS", ns);
FETCHPAR("NS", &noofscans);
fprintf(logfp, "after change, I got %d scans\n",
        noofscans);
// pull + set r.d.
FETCHPAR("D1", &repDelay);
```

```
fprintf(logfp, "I got d1: %f\n", repDelay);
STOREPAR("D1", my_d1);
FETCHPAR("D1", &repDelay);
fprintf(logfp, "after change, I got d1: %f\n", repDelay);
ZG_OVERWRITE;
ERRORABORT;
EFP;
APK;
//}}}

// {{{ loop for nutation curve
for (i = 0; i < 3; i++) {
  if (stillgoing > 0) {
    fprintf(logfp,
            "**********************************\n");
    fprintf(logfp,
            "Beginning point %i of nutation curve, the "
            "experiment "
            "will be in expno=%d...\n",
            i, expno + 1);
    FETCHPAR("P1", &p1);
    fprintf(logfp, "I had p1 of %f\n", p1);
    p1 = p1_list[i];
    fprintf(logfp, "I want p1 to be %f\n", p1);
    STOREPAR("P1", p1);
    FETCHPAR("P1", &p1);
    fprintf(logfp, "I set p1 to %f\n", p1);
    // pull + set number of scans
    FETCHPAR("NS", &noofscans);
    fprintf(logfp, "I got %d scans\n", noofscans);
    ns = 1;
    STOREPAR("NS", ns);
    FETCHPAR("NS", &noofscans);
    fprintf(logfp, "after change, I got %d scans\n",
            noofscans);
    // pull + set r.d.
    FETCHPAR("D1", &repDelay);
    fprintf(logfp, "I got d1: %f\n", repDelay);
    STOREPAR("D1", my_d1);
    FETCHPAR("D1", &repDelay);
    fprintf(logfp, "after change, I got d1: %f\n",
            repDelay);
    IEXPNO;
    SETCURDATA; // pg 15 -- IEXPNO changes current
                // dataset, but doesn't
    //             "make it available" -- must be
    //             followed by SETCURDATA for other
    //             commands that access the current
    //             dataset (I believe that here that
    //             includes our settitle function)
    // {{{ set processing params and title unique to
    // nutation
    sprintf(titlestr, "nutation step %d", i);
    settitle(titlestr);
    STOREPAR("LB", 5.0);
    STOREPAR("PSIGN", 2); // "both"
    // }}}
    ZG_OVERWRITE;
    EFP // exp FT and apply PHC0 and PHC1 -- don't think
        // it alters PHC0 and PHC1
        if (i == 0){
            APK // this alters PHC0 and PHC1
        } FETCHPAR("PSCAL", &pscal_save);
    STOREPAR("PSCAL", 6);
    fprintf(logfp, "pscal_save is %d\n", pscal_save);
    PP; // peak pick
    ERRORABORT;
    numPeaks = readPeakList(PROCPATH(0));
    fprintf(logfp, "I find %d peaks\n", numPeaks);
    // store the max peak intensity, frequency, and ppm
    // in maxip[s,h,p]
    maxips = 0.0;
    maxpsh = 0.0;
    for (j = 0; j < numPeaks; j++) {
      peakIntensity = getPeakIntensity(j);
      peakFreqHz = getPeakFreqHz(j);
      peakFreqPPM = getPeakFreqPPM(j);
      fprintf(logfp, "check peak intensity %f at %f\n",
              peakIntensity, peakFreqPPM);
      fprintf(logfp, "comparing %f to %f\n",
              abs(peakIntensity), abs(maxips));
      if (abs(peakIntensity) > abs(maxips)) {
        maxips = peakIntensity;
        maxpsh = peakFreqHz;
        maxpsp = peakFreqPPM;
      }
    }
    if (i == 0) {
      first_maxpsp = maxpsp;
    } else {
      if (abs(first_maxpsp - maxpsp) > 1.0) {
        fprintf(logfp, "I'm exiting because the "
                       "frequency deviation "
                       "is too large\n");
        stillgoing = 0;
      }
    }
  }
  if (stillgoing > 0) {
    fprintf(logfp,
            "for nutation step %d, the peak parameters "
            "are ips=%f "
            "psh=%f psp=%f\n",
            i, maxips, maxpsh, maxpsp);
    freePeakList();
    fprintf(logfp,
            "For my first nutation, I set p1 to %f\n",
            p1);
    fprintf(
        logfp,
        "For my first nutation, I get peak max of %f\n",
        maxips);
    nutation_peaks[i] = maxips;
  }
}
// }}}

// {{{ find the 90 time from the list of 3 peaks
if (stillgoing > 0) {
  fprintf(logfp,
          "*** *** *** *** *** *** *** *** ***\n");
  fprintf(logfp, "result of nutation curve:\n");
  for (i = 0; i < 3; i++) {
    fprintf(logfp, "p1=%f\theight=%f\n", p1_list[i],
            nutation_peaks[i]);
  }
  if (nutation_peaks[2] > 0) {
    fprintf(logfp, "I'm going to quit b/c the 3rd "
                   "nutation peak is positive!\n");
    stillgoing = 0;
  }
}
if (stillgoing > 0) {
  REAL m, b, r2;
  for (i = 0; i < 3; i++) {
    p1_list_forreg[i] = (REAL)p1_list[i];
  }
  linreg(3, p1_list_forreg, nutation_peaks, &m, &b, &r2);
  p1 = -b / m / 2.;
  IEXPNO;
```

```
  fprintf(logfp, "m=%f b=%f r=%g\n", m, b, r2);
  fprintf(logfp, "going to set p1 to %f in %d\n", p1,
          expno);
  STOREPAR("P1", p1);
  ZG_OVERWRITE;
  ERRORABORT;
  EFP;
  APK;
  if (p1 > 800 || p1 < 0) {
    fprintf(logfp, "that's an invalid value for "
                   "p1!!!\nI'm going to quit!!!\n");
    STOREPAR("P1", 360.0);
    stillgoing = 0;
  }
}
// }}}

if (stillgoing) {
  //{{{ 2H IR
  IEXPNO
  SETCURDATA
  fprintf(logfp, "**********************************\n");
  fprintf(logfp,
          "Incrementing experiment number to %d...\n",
          expno);
  RPAR("ab_2H_T1_logSpace", "all");
  STOREPAR("SFO1", sfo1);
  STOREPAR("O1", o1);
  FETCHPAR("SFO1", &sfo1);
  FETCHPAR("O1", &o1);
  fprintf(logfp, "In IR expjeriment, I set SFO1 to %f\n",
          sfo1);
  fprintf(logfp, "In IR experiment, I set O1 to %f\n",
          o1);
  FETCHPAR1("SFO1", &sfo1);
  FETCHPAR1("O1", &o1);
  fprintf(
      logfp,
      "In IR experiment, I have SFO1 (indirect) as %f\n",
      sfo1);
  fprintf(
      logfp,
      "In IR experiment, I have O1 (indirect) as %f\n",
      o1);
  STOREPAR("P1", p1);
  fprintf(logfp, "In IR experiment, I set P1 to %f\n",
          p1);
  FETCHPAR("P1", &p1);
  fprintf(logfp, "In IR experiment, I get P1 of %f\n",
          p1);
  // pull + set r.d.
  FETCHPAR("D1", &repDelay);
  fprintf(logfp, "I got d1: %f\n", repDelay);
  STOREPAR("D1", my_d1);
  FETCHPAR("D1", &repDelay);
  fprintf(logfp, "after change, I got d1: %f\n",
          repDelay);
  ZG_OVERWRITE;
  //}}}
}
// close down

fclose(logfp);
QUIT

// the above code relies on some standard C functions,
// which rely on other (standard) headers: these are
// defined here.
//
// Note that we do need to include the function
// declarations at the top of the AU file, as well.

#include <math.h>
#include <stdlib.h>

    inline static REAL
    sqr(REAL x) {
  return x * x;
}
// linear regression function
int linreg(int n, const REAL x[], const REAL y[],
           REAL *m, REAL *b, REAL *r) {
  REAL sumx = 0.0;  /* sum of x     */
  REAL sumx2 = 0.0; /* sum of x**2  */
  REAL sumxy = 0.0; /* sum of x*y   */
  REAL sumy = 0.0;  /* sum of y     */
  REAL sumy2 = 0.0; /* sum of y**2  */
  int i;

  for (i = 0; i < n; i++) {
    sumx += x[i];
    sumx2 += sqr(x[i]);
    sumxy += x[i] * y[i];
    sumy += y[i];
    sumy2 += sqr(y[i]);
  }

  REAL denom = (n * sumx2 - sqr(sumx));
  if (denom == 0) {
    // singular matrix. can't solve the problem.
    *m = 0;
    *b = 0;
    if (r)
      *r = 0;
    return 1;
  }

  *m = (n * sumxy - sumx * sumy) / denom;
  *b = (sumy * sumx2 - sumx * sumxy) / denom;
  if (r != NULL) {
    *r =
        (sumxy - sumx * sumy /
                     n) / /* compute correlation coeff */
        sqrt((sumx2 - sqr(sumx) / n) *
             (sumy2 - sqr(sumy) / n));
    double slope = ((n * sumxy) - (sumx * sumy)) / denom;
    double intercept =
        ((sumy * sumx2) - (sumx * sumxy)) / denom;
    double term1 = ((n * sumxy) - (sumx * sumy));
    double term2 = ((n * sumx2) - (sumx * sumy));
    double term3 = ((n * sumy2) - (sumy * sumy));
    double term23 = (term2 * term3);
    double r2 = 1.0;
    if (fabs(term23) > MIN_DOUBLE)
      r2 = (term1 * term1) / term23;
  }
  return 0;
}

void settitle(char *string) {
  char titlename[1000];
  FILE *titlefp;
  (void)sprintf(titlename, "%s/%s/%d/pdata/%d/title",
                disk, name, expno, procno);
  if ((titlefp = fopen(titlename, "wt")) == NULL) {
    Proc_err(DEF_ERR_OPT, "Can't open %s\n%s", titlename,
             strerror(errno));
    return 0;
  }
  fprintf(titlefp, string);
```

```
  fclose(titlefp);
}
```

The Bruker IconNMR program was configured with the shimming option `lctshim` coupled with specialized solvent configured via `topshim solvcal` (Tol-H8 as starting point, with ^{1}H shim nucleus and manual `o1p=2`.)

B. Pulse Program

The pulse program is a standard inversion recovery, but employs (1) utilization of the lock channel for acquisition and (2) separately stored phase cycles following the recipe given previously.[S13]

```
;ab_IR2h (written aug2020)
;based off egr_IR
;avance-version (12/01/11)
;1D sequence
;using 2H lockswitch unit or BSMS 2H-TX board
;
;£CLASS=HighRes
;£DIM=1D
;£TYPE=
;£SUBTYPE=
;£COMMENT=


#include<Avance.incl>
#include<Sysconf.incl>

define loopcounter total_ph_steps

"p2=p1*2"
"d11=30m"
"acqt0=-p1*2/3.1416"
"l20=2" ;steps in ph1
"l21=4" ;steps in ph2
"total_ph_steps = l20*l21"
"l23=td1/total_ph_steps"
;td1=number of phase steps * vdlist
;td2=4096

1 ze
  d11 LOCKDEC_ON
  d11 H2_PULSE

2 30m rpp1
  1m rpp2

3       30m H2_LOCK
        d1
        d11 H2_PULSE
        p2:D ph1
        vd
        p1:D ph2
        goscnp ph31
        d11 wr #0 if #0
        2u ipp2
      lo to 3 times l21
    2u ipp1
    lo to 3 times l20
  0.1u ivd
  lo to 2 times l23
  d11 H2_LOCK
  d11 LOCKDEC_OFF
exit


ph1=0 2
ph2=0 1 2 3
ph31=0


;pl1 : f1 channel - power level for pulse (default)
;p1 : f1 channel -  90 degree high power pulse
;d1 : relaxation delay; 1-5 * T1
;d11: delay for disk I/O                              [30 msec]
;ns: 1 * n, total number of scans: NS * TD0

;locnuc: off



;£Id: zg2h,v 1.14.8.1 2012/01/31 17:56:41 ber Exp £
```

S11. Inverse Laplace Transform

A PDF export of an explanatory jupyter notebook that converts raw data from these programs to a correlated distribution of T_1 *vs.* chemical shift is attached at the end of this supporting information document. (The data in the resulting HDF file is read into subsequent scripts that compile and display contours from multiple experiments.)

[S1] M. Ueda and Z. A. Schelly. "Mean aggregation number and water vapor pressure of AOT reverse micellar systems determined by controlled partial pressure-vapor pressure osmometry (CPP-VPO)." *Langmuir*, 4(3):653–655 (1988) doi:10.1021/la00081a026.

[S2] H.-F. Eicke. "Surfactants in nonpolar solvents." In "Micelles," volume 87, pages 85–145. Springer-Verlag, Berlin/Heidelberg (1980). ISBN 978-3-540-09639-9.

[S3] I. R. Piletic, D. E. Moilanen, D. B. Spry, N. E. Levinger, and M. D. Fayer. "Testing the core/shell model of nanoconfined water in reverse micelles using linear and nonlinear ir spectroscopy." *J. Phys. Chem. A*, 110(15):4985–4999 (2006) doi:10.1021/jp061065c.

[S4] A. Abragam. *The Principles of Nuclear Magnetism*, pages 314–315. Clarendon Press (1961). ISBN 978-0-19-852014-6. Google-Books-ID: 9M8U_JK7K54C.

[S5] C. P. Slichter. *Principles of Magnetic Resonance*, pages 163–171. Springer-Verlag, third edition (1989).

[S6] B. P. Wiebenga-Sanford, J. DiVerdi, C. D. Rithner, and N. E. Levinger. "Nanoconfinement's Dramatic Impact on Proton Exchange between Glucose and Water." *J. Phys. Chem. Lett.*, 7(22):4597–4601 (2016) doi:10.1021/acs.jpclett.6b01651.

[S7] B. S. Marques, N. V. Nucci, I. Dodevski, K. W. C. Wang, E. A. Athanasoula, C. Jorge, and A. J. Wand. "Measurement and Control of pH in the Aqueous Interior of Reverse Micelles." *J. Phys. Chem. B*, 118(8):2020–2031 (2014) doi:10.1021/jp4103349.

[S8] P. Honegger and O. Steinhauser. "Hydration dynamics of proteins in reverse micelles probed by 1 H-NOESY/ 1 H-ROESY NMR and 17 O-nuclear quadrupole resonance (NQR)." *Phys. Chem. Chem.*

Phys., 21(27):14571–14582 (2019). PMID: 31237595 doi:10.1039/C9CP02654A.

[S9] W. D. Van Horn, M. E. Ogilvie, and P. F. Flynn. "Reverse Micelle Encapsulation as a Model for Intracellular Crowding." *J. Am. Chem. Soc.*, 131(23):8030–8039 (2009) doi:10.1021/ja901871n.

[S10] P. Marzola, C. Pinzino, and C. A. Veracini. "Spin-labeling study of human serum albumin in reverse micelles." *Langmuir*, 7(2):238–242 (1991) doi:10.1021/la00050a006.

[S11] B. G. Lefebvre, W. Liu, R. W. Peterson, K. G. Valentine, and A. J. Wand. "NMR spectroscopy of proteins encapsulated in a positively charged surfactant." *J. Magn. Reson.*, 175(1):158–162 (2005) doi:10.1016/j.jmr.2005.03.008.

[S12] B. Fuglestad, B. S. Marques, C. Jorge, N. E. Kerstetter, K. G. Valentine, and A. J. Wand. "Chapter Two - Reverse Micelle Encapsulation of Proteins for NMR Spectroscopy." In A. J. Wand, editor, "Methods in Enzymology," volume 615 of *Biological NMR Part B*, pages 43–75. Academic Press (2019).

[S13] A. A. Beaton, A. Guinness, and J. M. Franck. "A modernized view of coherence pathways applied to magnetic resonance experiments in unstable, inhomogeneous fields." *J. Chem. Phys.*, 157(17):174204 (2022) doi:10.1063/5.0105388.
