# Supplementary material for: Rapidly Screening the Correlation between the Rotational Mobility and the Hydrogen Bonding Strength of Confined Water
Source: J Phys Chem B. 2024 Oct 23;128(43):10749–63. doi: 10.1021/acs.jpcb.4c05397 (PMC11533181; doi:10.1021/acs.jpcb.4c05397)
Supplement: Supplementary file 2 — jp4c05397_si_002.pdf [file jp4c05397_si_002.pdf]

"Rapidly Screening the Correlation Between the Rotational Mobility and the Hydrogen Bonding Strength of Confined Water" (Supporting Information ⇒ Accompanying Jupyter Notebook)

Alec A. Beaton   Alexandria Guinness   John M. Franck

September 22, 2024

The only thing that we need to import is the following, which relies on the freely available pySpecData library supplied by the authors (source code and installation instructions here).

Note that one important consequence of this is that when you use jupyter notebook to "inspect" (include a variable/object at the end of a cell on its own line) a pySpecData `nddata` object, it will typically generate an automatically chosen, appropriate plot.

[1]:
```
%load_ext pyspecdata.ipy
```

Specify name of NMR dataset and associated experiment number of T experiment. This could easily be extend to a loop over a list of different dataset names and/or experiment numbers to enable processing of multiple datasets in one script.

[2]:
```
exp_name = "RM1_nov23_2H_211201au"
expno = 7
```

Use pySpecData to locate the data (`exp_type` gives the location of directories that are registered in the `.pyspecdata` or `_pyspecdata` configuration file in the user's home directory) Note that, in the normal course of things, we achieve this through ensuring the following line in `~/.pyspecdata`:

```
[General]
data_directory = /home/jmfranck/exp_data
[RcloneRemotes]
nmr_data_aab = jmf_teams:General/exp_data/NMR_Data_AAB/
```

where the square brackets indicate sections to the config file (these are likely already present), where the directory `/home/jmfranck/exp_data/NMR_Data_AAB` exists on the local computer, and where the `rclone` command-line tool has been pre-configured so that `jmf_teams` points to a particular cloud resource containing the laboratory's data (e.g. here, a microsoft teams/sharepoint share.) For optimal speed, the data is copied once to the local computer upon first running the script, after which it is available offline. Note that the point of this mechanism is that each individual user can select the location of `/home/jmfranck/exp_data` where experimental data is copied, and can also point to multiple rclone remotes that can be, *e.g.* google drive, as well as sharepoint, onedrive, etc. Note that pySpecData reads zipped directories of Bruker data (which is the recommended way to store Bruker data).

[3]:
```
d = find_file(
    exp_name, exp_type="NMR_Data_AAB", dimname="indirect", expno=expno
)
d.setaxis("indirect", None)
# semicolon supresses notebook "inspection", which here would display a plot of
 ↪the data
```

```
warning: ignoring second dimension SFO1, since it's probably wrong
subpath is ('7', 'vdlist')
```

[3]:

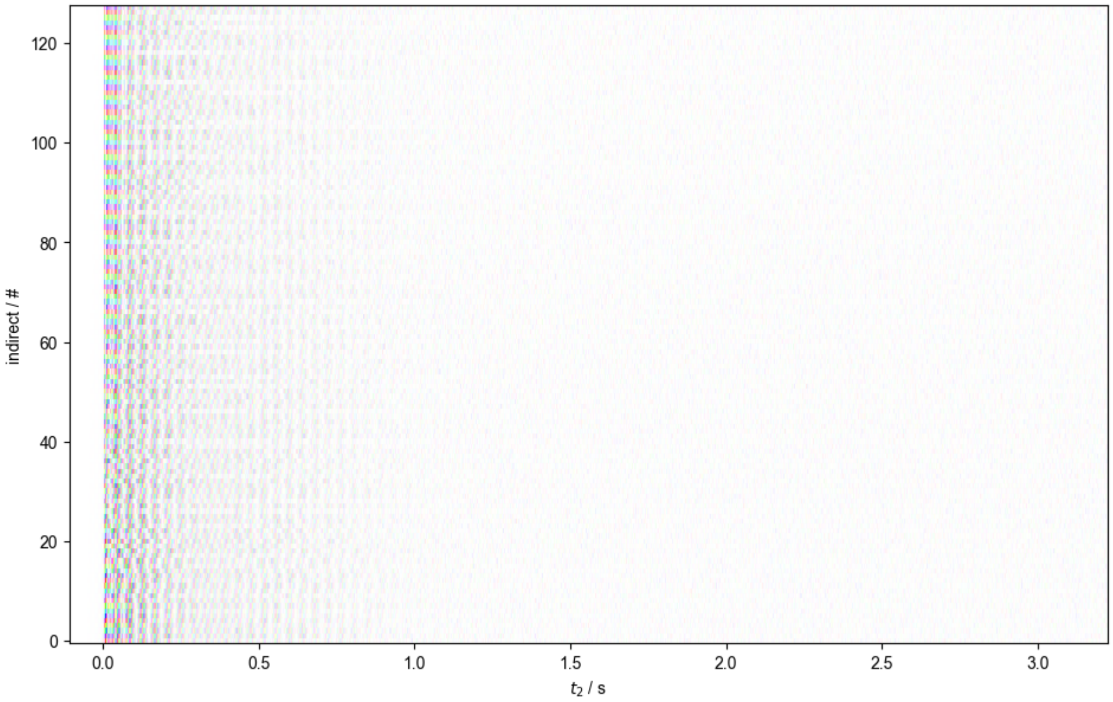

Pull relevant parameters from Bruker dataset

[4]:
```
l20 = d.get_prop("acq")["L"][20]
l21 = d.get_prop("acq")["L"][21]
SFO1 = d.get_prop("acq")["SFO1"]
O1 = d.get_prop("acq")["O1"]
BF1 = d.get_prop("acq")["BF1"]
vd_list = d.get_prop("vd")
SFO1
```

[4]: 61.422692237149

0.1 Prepare the relevant multidimensional axes for data processing

The supplied pulse sequence stores the phase cycle along the indirect dimension, along with the changing relaxation delay. (see Beaton *et. al.* JCP 2022) We use pySpecData `chunk` to separate the information into 3 appropriately named dimensions.

[5]:
```
d.chunk("indirect", ["indirect", "ph1", "ph2"], [-1, 120, 121]);
```

We label the phase cycling dimensions appropriately

[6]:
```
d.setaxis("ph1", r_[0, 2.0] / 4)
d.setaxis("ph2", r_[0:4.0] / 4);
```

and the same for the indirect

[7]:
```
d.setaxis("indirect", vd_list)
d.rename("indirect", "vd");
```

Reorder the dimensions; this is mostly for the purposes of plotting. Python uses "c-ordering" meaning that the dimensions listed first are furthest apart in memory – *i.e.* if the data were read with nested loops, this would be the outermost loop, while the rightmost dimension would be the innermost loop. Here, we omit the final semicolon in order to look at the resulting data. (See Beaton *et. al.* to understand the data representation given here. The version given here is a compact version of the plots described in the paper, with the y axis giving the size and axis ranges of the lexicographically ordered dimensions.).

[8]:
```
d.reorder(["ph1", "ph2", "vd"])
```

[8]:

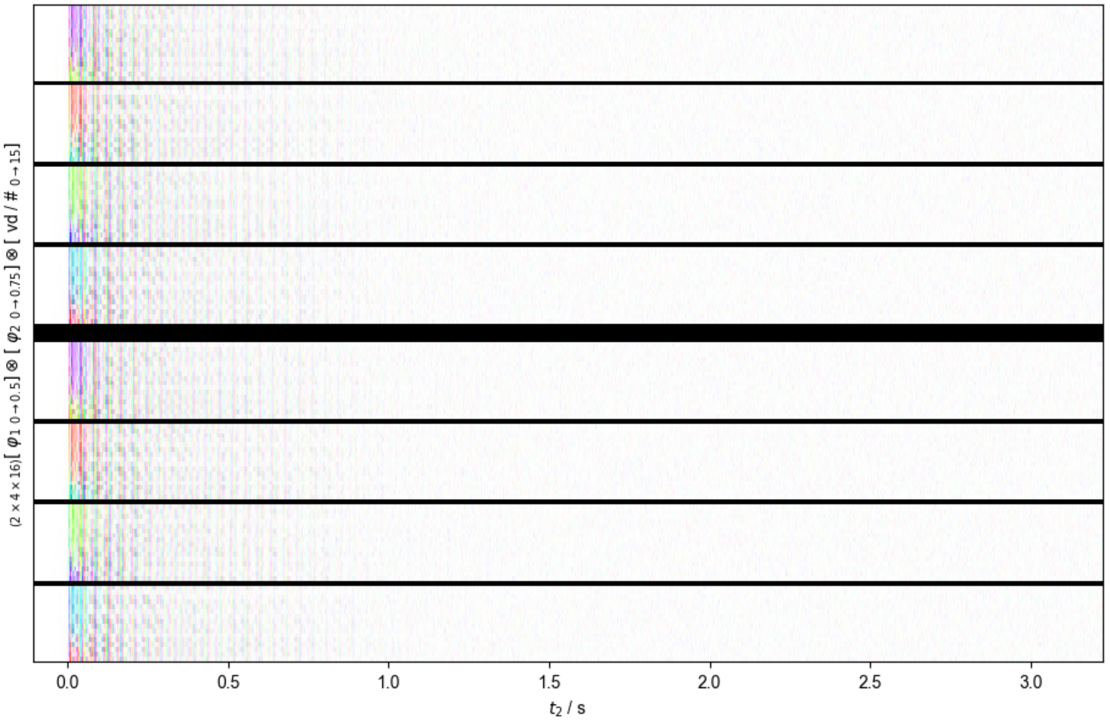

Apply fourier transformation, from t_2 to ν_2 as well as along the phase cycling dimensions, to convert ϕ_j to Δp_j, in the coherence transfer domain (see Beaton *et. al.* JCP 2022)

We see that the data shows up in the expected coherence pathway, $\Delta p_1 = 0$, $\Delta p_2 = -1$. We are also able to see that the phase cycling successfully removes artefactual pathways that arise from isooctane solvent (two peaks near $\nu_2 = -200$ Hz) that is partially excited by the first pulse; this likely arises from magnetization from the long-relaxing solvent that has not fully returned to equilibrium. Finally, we are able to see that all the polarization that is excited from the water appears entirely in the desired pathway (none is lost to artefactual pathways).

[9]:
```
d.ft("t2", shift=True)
d.ft(["ph2", "ph1"], unitary=True)
```

[9]:

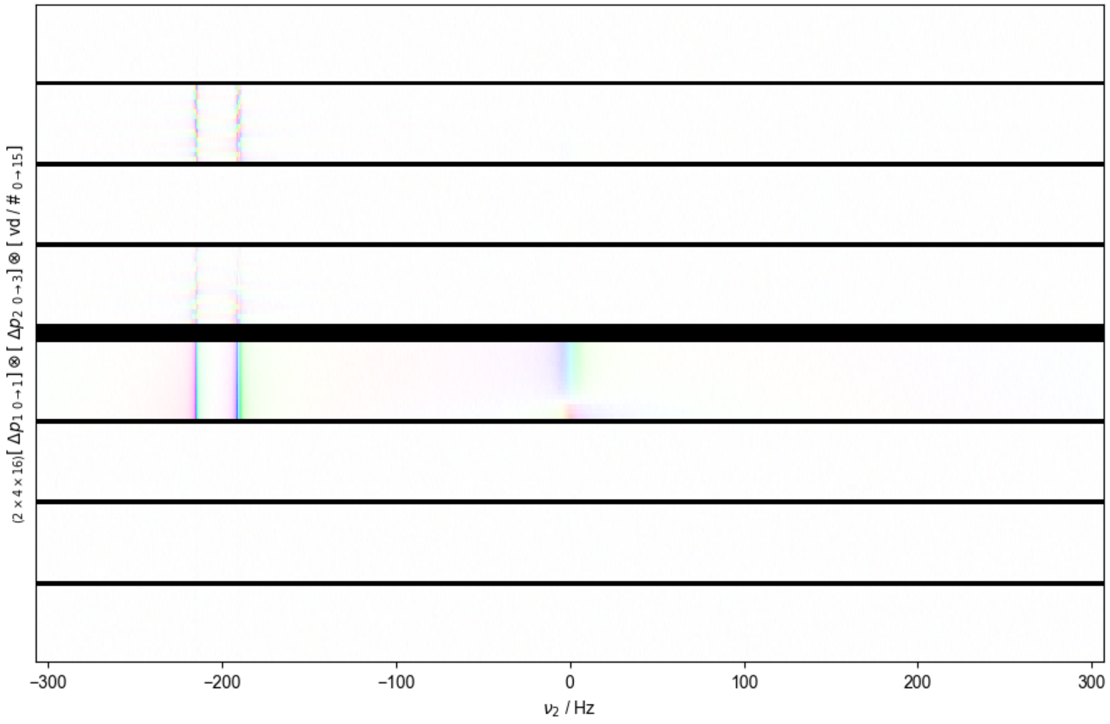

[10]:
```
coh_sel = {"ph1": 0, "ph2": -1}
```

We shift back to the time domain, in order to correct for the group delay and obtained phased spectra. Note that pySpecData will "remember" the starting point of our FT, and upon subsequent FT will will apply an appropriate phase shift to account for changes to the x-axis.

```
[11]: d.ift("t2")
with figlist_var() as fl:
    fl.next("illustrate timing correction")
    fl.plot(
        d["t2":(None, 0.010)]["vd", -1]["ph2", coh_sel["ph2"]][
            "ph1", coh_sel["ph1"]
        ]
    )
    fl.plot(
        abs(d)["t2":(None, 0.010)]["vd", -1]["ph2", coh_sel["ph2"]][
            "ph1", coh_sel["ph1"]
        ]
    )
    time_of_max = (
        abs(d)[
            "t2":(
                None,
                0.005,
            )  # select up to 5 ms (see pySpecData documentation on how we␣
↪enable fancy slicing styles)
        ][
            "vd",
            -1,  # select the last element of the T1 indirect dimension (where␣
↪data has fully recovered
        ][
            "ph2", coh_sel["ph2"]
        ][
            "ph1",
            coh_sel["ph1"],  # select the coherence pathway given by coh_sel
        ]
        .argmax("t2")
        .item()
    )  # find the value of t2 where the result rises to a max, and (item) give␣
↪it as a float
    axvline(x=time_of_max * 1e3, linestyle=":", color="black")
    # time_of_max = 0.004884
    d.setaxis("t2", lambda x: x - time_of_max)
```

```
1: illustrate timing correction |||ms
```

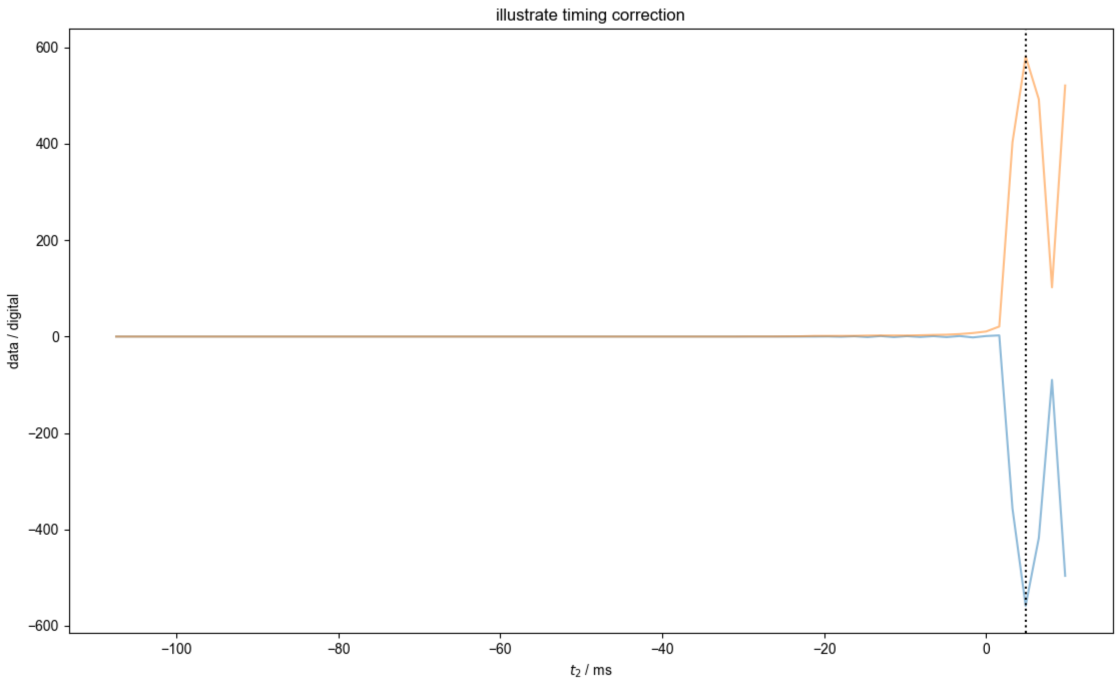

We find that slicing a clean FID (causal signal) is a simple technique that generates a relatively baseline-free signal (*i.e.* we are eliminating some initial points preceding $t = 0$ of the FID that are artefacts of the of the oversampling/filtering process. This process is not idealized, but is fast and robust in this context.

[12]:
```
d = d["t2":(0, None)]
d["t2":0] *= 0.5
```

Specify region of interest for ^{2}H peaks. First, grab the coherence pathway of interest, and convert to ppm:

[13]:
```
d_sliced = d["ph1", coh_sel["ph1"]]["ph2", coh_sel["ph2"]].ft("t2").to_ppm()
d_sliced  # inspect
```

[13]:

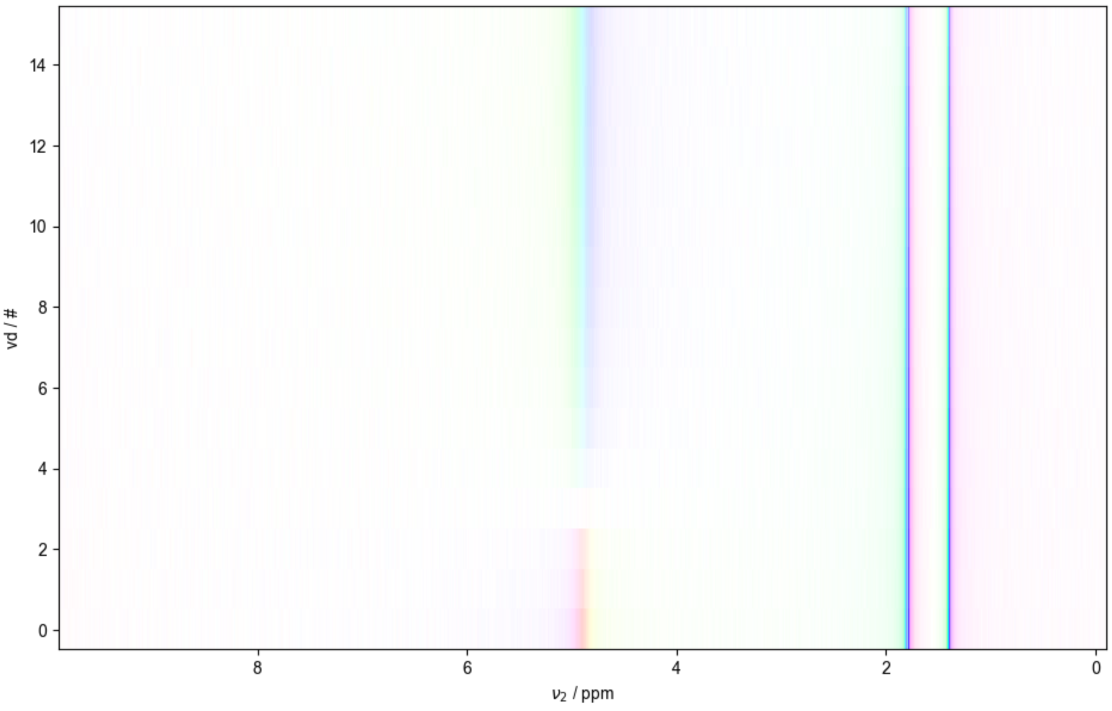

Give good tuple for where water peak resides in ppm for chemical shift referencing

[14]:
```
water_peak = (4, 6)
```

Now, go ahead and slice out the water peak:

[15]:
```
d_sliced = d_sliced["t2":water_peak]  # slice from 4 to
# 6 ppm.  Note that currently, pySpecData continues to
# use the same name for the dimension, in both the
# frequency and time domains.  Addition of the ability to
# use, e.g.   is expected in a future version.
d_sliced
```

[15]:

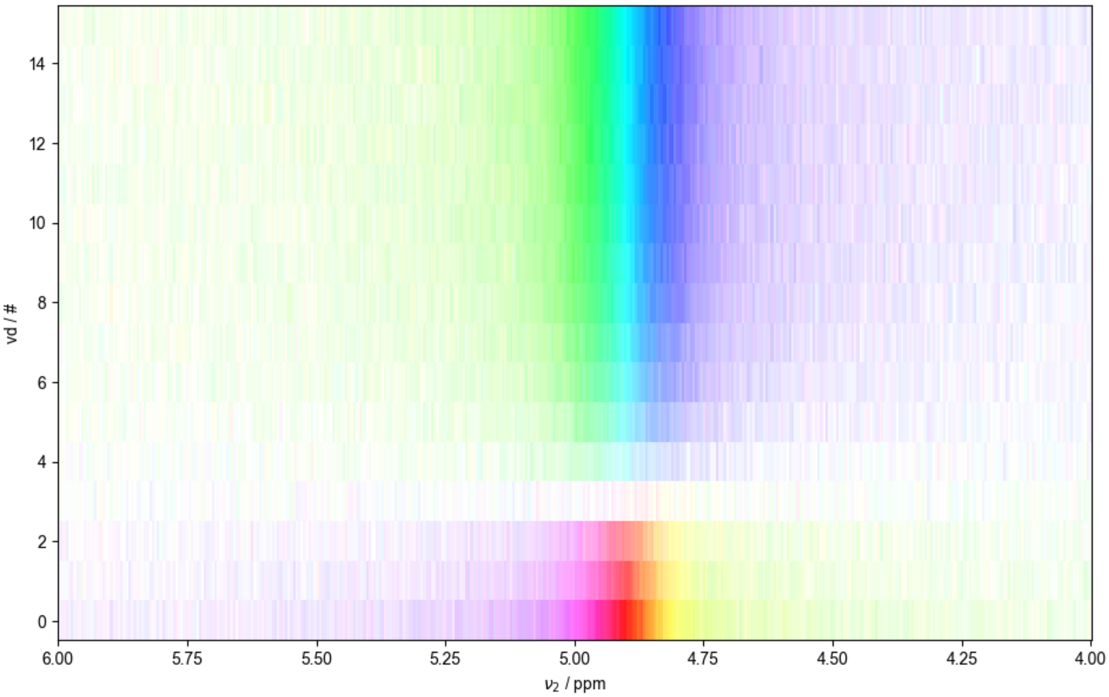

Note that in order to be sure to catch fast-relaxing components, the first few points of the inversion recover variable delay (`vd`) list are spaced more closely:

[16]:
```
figure(figsize=(4, 2))
plot(d.getaxis("vd"), "o")
print(d.getaxis("vd")[:2])
xlabel("position in list")
```

```
[0.0001 0.0114]
```

```
/home/jmfranck/git_repos/pyspecdata/pyspecdata/core.py:987: UserWarning: marker
is redundantly defined by the 'marker' keyword argument and the fmt string "o"
(-> marker='o'). The keyword argument will take precedence.
  retval = myplotfunc(*plotargs,**kwargs)
```

[16]:
```
Text(0.5, 0, 'position in list')
```

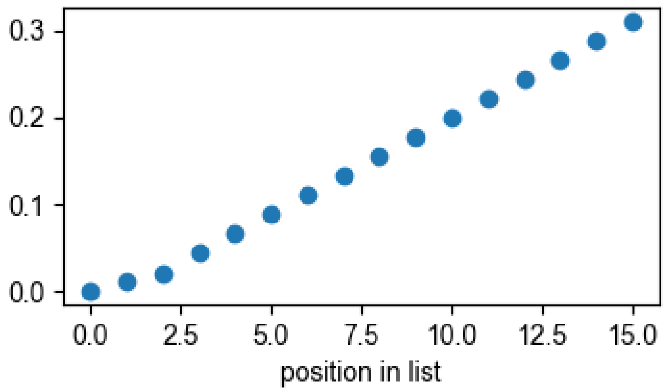

So, we can use a pcolormesh to look at the unevenly spaced data:

[17]:
```
d_sliced.pcolor()
```

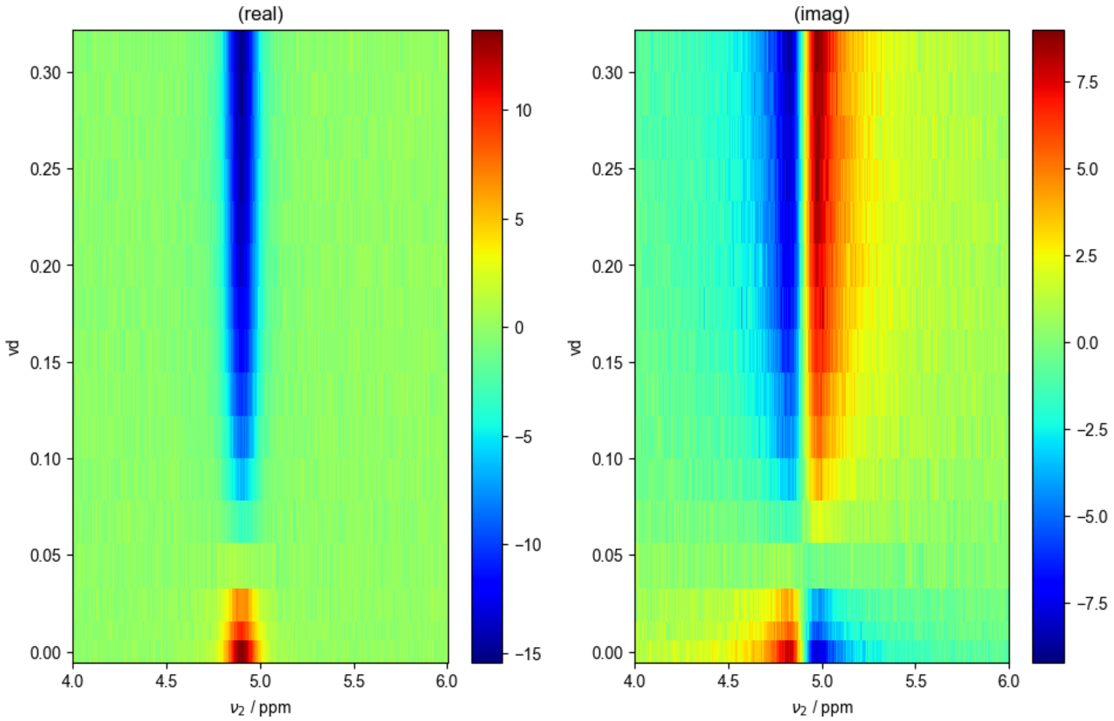

ppm_setting is determined by a separate ^{1}H measurement for the same sample referencing the water peak observed in the ^{1}H spectrum relative to TMS added to the oil phase of the RM mixture

[18]:
```
actual_water_ppm = 4.54
```

[19]:
```
current_water_ppm = (
    abs(d_sliced)["vd", -1].argmax("t2").item()
)  # pull the last (fully relaxed) signal in the variable delay list
print("Chemical shift was", current_water_ppm)
d_sliced["t2"] -= current_water_ppm  # shift axes to remove current water ppm
d_sliced["t2"] += actual_water_ppm  # and add the actual shift
this_nu = abs(d_sliced)["vd", -1].argmax("t2").item()
print("After correcting, chemical shift is", this_nu)
fl.next("peak pick")
fl.plot(d_sliced["vd", -1])
fl.plot(d_sliced["vd", -1].imag)
axvline(actual_water_ppm, linestyle=":")
```

```
Chemical shift was 4.896774085022195
After correcting, chemical shift is 4.54
```

[19]: `<matplotlib.lines.Line2D at 0x7ff7a6e656d0>`

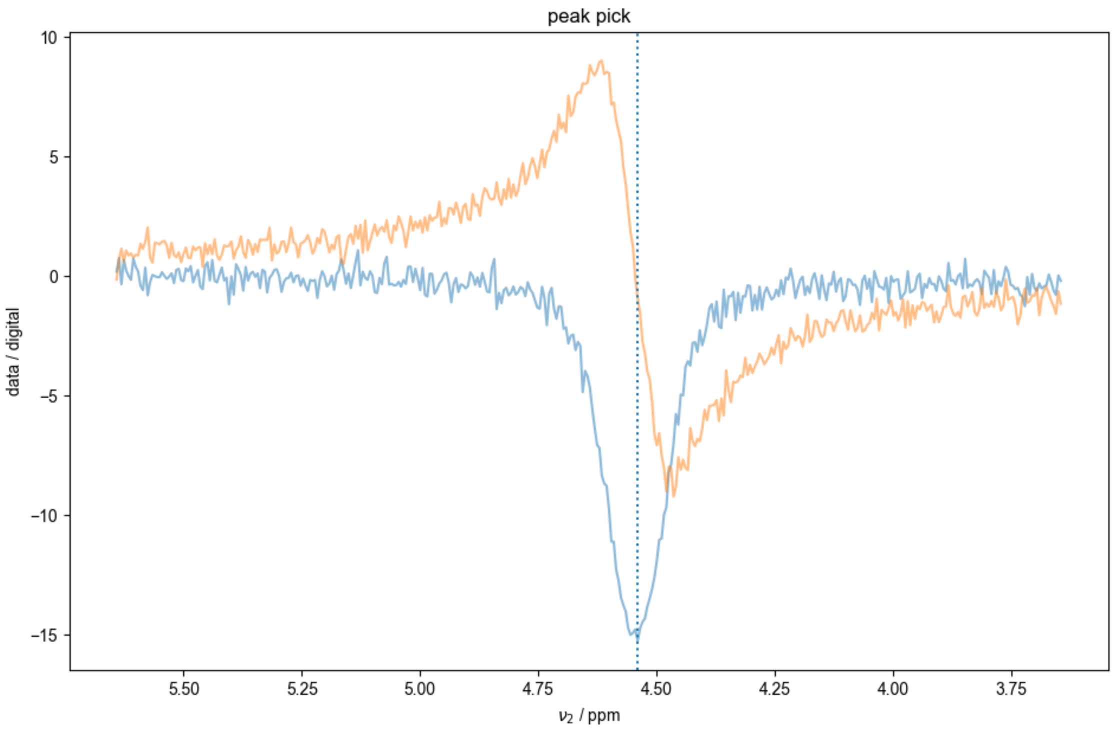

to optimize SNR without distortion, we apply a Gaussian apodization window with $\sigma = 0.0661$ Hz

[20]:
```
d_sliced.ift("t2")  # move into the time domain
filter_width = 0.0661 * SFO1
d_sliced *= exp(-d_sliced.fromaxis("t2") ** 2 / 2 / filter_width**2)
d_sliced.ft("t2")
```

```
d_sliced.pcolor()
```

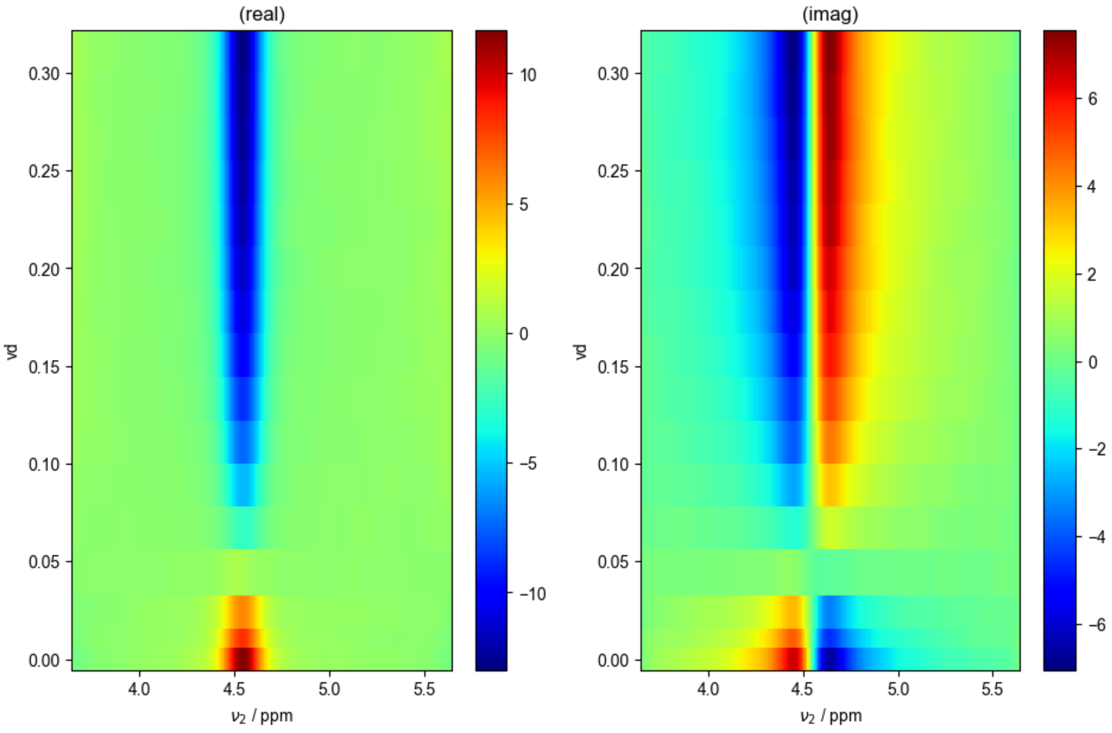

Prepare data for ILT, perform ILT, and save as h5

[21]:
```
nd_data = d_sliced
nd_data *= -sign(
    nd_data["vd":0.0].real.sum("t2").item()
)  # make sure the first vd scan is inverted (negative), since methods above␣
 ↪aren't sensitive to this
nd_data.real
```

[21]:

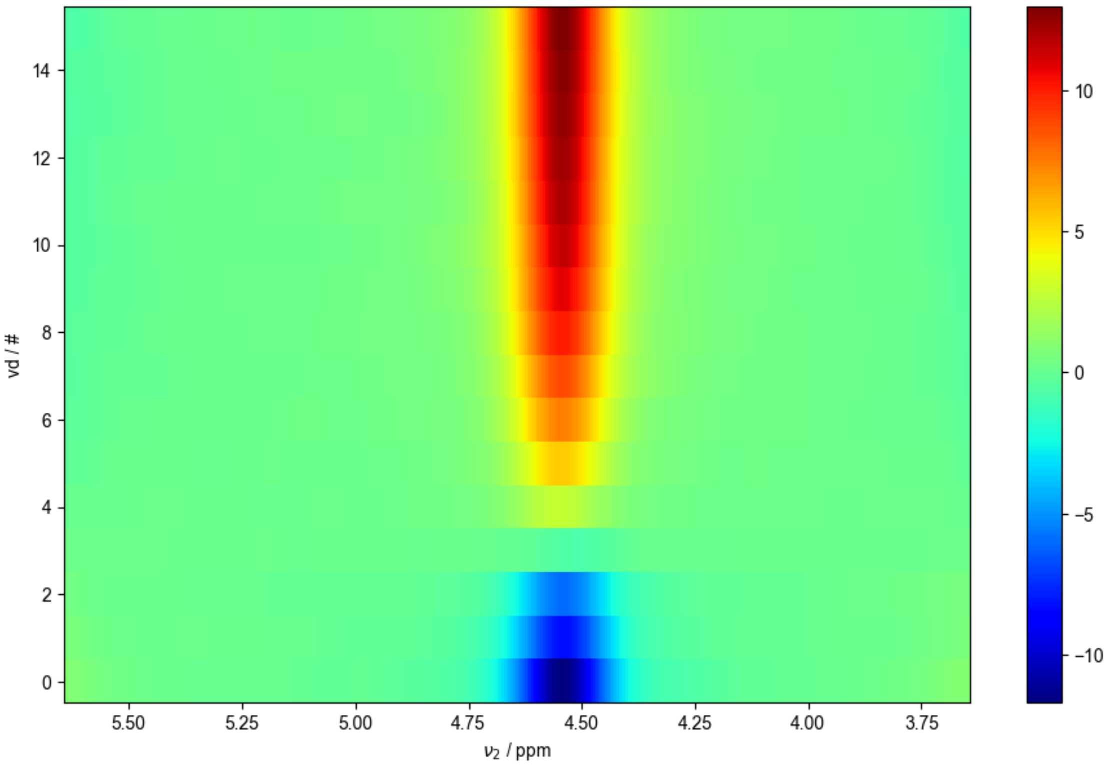

[22]:
```
logT1 = nddata(
    r_[
        log10(
            nd_data.getaxis("vd")[1]
        ) : log10(  # base the lower bound of the T basis (shortest decay␣
↪constant) on the second point
            0.75
        ) : 150j
    ],
    "logT1",
)
# ndshape(...).alloc() provides an object-oriented alternative to numpy "zeros"
nd_soln = ndshape(
    [logT1.data.size, nd_data.shape["t2"]], ["logT1", "t2"]
).alloc(dtype=float64)
nd_soln.copy_props(nd_data)  # copies experimental parameters, etc. of data
nd_soln.setaxis("t2", nd_data.getaxis("t2"));
```

Next, we tell the pySpecData **nnls** routine to perform non-negative regularization of the data, using the basis function $1 - 2\exp(-\mathrm{vd}/10^y$, where y is the output dimension (corresponding to $y = \log(T_1)$). The **nddata** object **T1** supplies the new $y = \log(T_1)$ axis (which is also used to compute the regularization kernel).

First, we run with a fixed regularization parameter. This runs very fast, and gives us a sense of what the data looks like, although the data here will be over or under-regularized.

[23]:
```
nd_data.real.C.nnls(
    "vd", logT1, lambda x, y: 1.0 - 2.0 * exp(-x / 10 ** (y)), l=3
)
```

[23]:

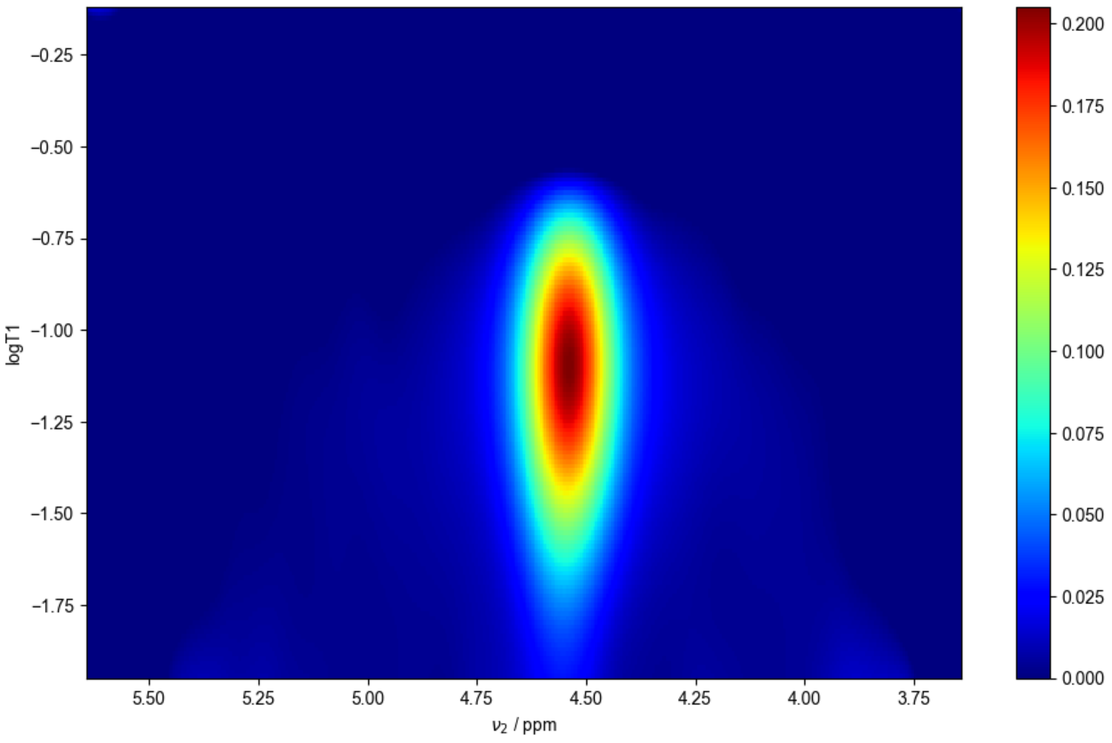

Next, we allow the BRD algorithm to choose the regularization parameter for each offset, and save the resulting data. Note that the final line in this cell (which writes the data to a file) will fail with an error if the data already exists (*i.e.*, it will refuse to just blindly wipe out previous data). If this happens when running this example, you can delete the output file (or use h5py to delete only the corresponding node).

[24]:
```
for t2_index, t2_point in enumerate(nd_data.getaxis("t2")):
    q = nd_data["t2", t2_index].real.C.nnls(
        "vd", logT1, lambda x, y: 1.0 - 2.0 * exp(-x / 10 ** (y)), l="BRD"
    )
    nd_soln["t2", t2_index]["logT1", :] = q
    if t2_index == 0:
        nd_soln.setaxis("logT1", q.getaxis("logT1"))
figure()
title("%s + expno %s APOD" % (exp_name, expno))
```

```
image(nd_soln)
nd_soln.name("nnls")
nd_soln.hdf5_write(
    "processed_" + exp_name + "_" + str(expno) + ".h5", getDATADIR("processed")
)
```

```
/home/jmfranck/git_repos/pyspecdata/pyspecdata/core.py:4365: RuntimeWarning:
divide by zero encountered in divide
  dangle_dA = 1/(2*self.data)
/usr/local/lib/python3.9/dist-packages/tables/atom.py:362: FlavorWarning:
support for unicode type is very limited, and only works for strings that can be
cast as ascii
  warnings.warn("support for unicode type is very limited, and "
```

```
WARNING!!, attributes ['other_info'] are dictionaries!
WARNING!!, attributes ['dimlabels'] are lists!
```

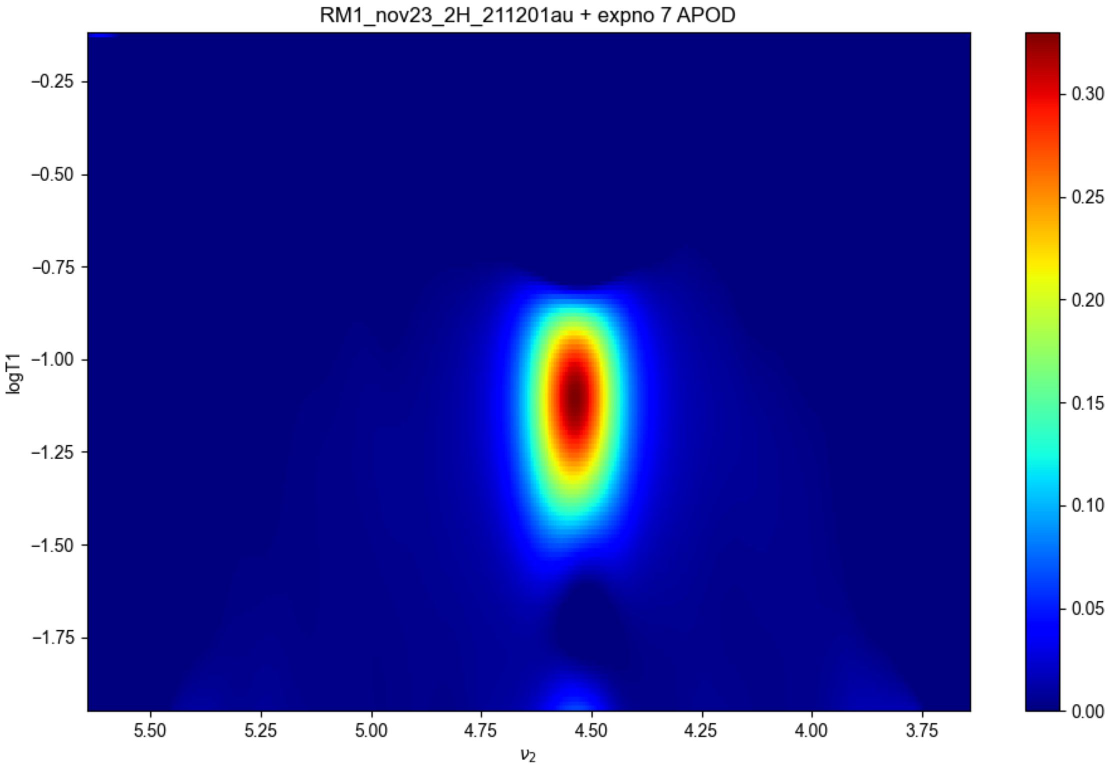

Note that the edge artefact observed for very fast relaxation times is a common ILT artefact (representing relaxation rates that are faster than the spacing between the points in the inversion recovery curve), and moves to even faster relaxation times as the basis is expanded.
